# Effect of Lanthanum‐Aluminum Co‐Doping on Structure of Hafnium Oxide Ferroelectric Crystals

**DOI:** 10.1002/advs.202410765

**Published:** 2024-12-04

**Authors:** Zhenhai Li, Shuqi Tang, Tianyu Wang, Yongkai Liu, Jialin Meng, Jiajie Yu, Kangli Xu, Ruihong Yuan, Hao Zhu, Qingqing Sun, Shiyou Chen, David Wei Zhang, Lin Chen

**Affiliations:** ^1^ School of Microelectronics, Fudan University State Key Laboratory of Integrated Chips and Systems Shanghai 200433 P. R. China; ^2^ School of Integrated Circuits Anhui University Hefei 230601 P. R. China; ^3^ School of Integrated Circuits, State Key Laboratory of Crystal Materials Shandong University Jinan 250100 P. R. China; ^4^ National Integrated Circuit Innovation Center Shanghai 201203 P. R. China; ^5^ Shaoxin Laboratory Shaoxing 312000 P. R. China

**Keywords:** 3D macaroni architecture, Al and La co‐doping, ferroelectricity, hafnium‐based device, high endurance cycles

## Abstract

Hafnium oxide (HfO_2_)‐based devices have been extensively evaluated for high‐speed and low‐power memory applications. Here, the influence of aluminum (Al) and lanthanum (La) co‐doping HfO_2_ thin films on the ferroelectric characteristics of hafnium‐based devices is investigated. Among devices with different La/Al ratios, the Al and La co‐doped hafnium oxide (HfAlAO) device with 4.2% Al and 2.17% La exhibited the excellent remanent polarization and thermostability. Meanwhile, first principal analyses verified that hafnium‐based thin films with 4.2% Al and 2.17% La promoted the formation of the o‐phase against the paraelectric phase, providing theoretical support for supporting experimental results. Furthermore, a vertical ferroelectric HfO_2_ memory based on 3D macaroni architecture is reported. The devices show excellent ferroelectric characteristics of 22 µC cm^−2^ under 4.5 MV cm^−1^ and minimal coercive field of ≈1.6 V. In addition, the devices exhibit great memory performance, including the response speed of device can achieve 20 ns and endurance characteristic can achieve 10^10^ cycles.

## Graphical abstract

The influence of Al and La co‐doping HfO_2_ thin films on the ferroelectric characteristics of hafnium‐based devices is investigated. Among devices with different La/Al ratios, the HfAlAO device with 4.2% Al and 2.17% La exhibited the excellent remanent polarization and thermostability. The first principal analyses verified that Al and La co‐doped hafnium‐based thin films promoted the formation of the o‐phase.

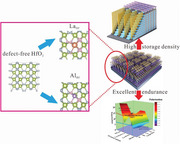

## 1 Introduction

With the rapid development of the artificial intelligence, the memory market is currently in an era of tremendous growth.^[^ ^1^, ^2^ ^]^ The big data explosion has yielded demand of more and more data storage.^[^ ^3^, ^4^, ^5^, ^6^ ^]^ Although 3D‐NAND memory is the high storage density,^[^ ^7^, ^8^ ^]^ it faces the challenge of some drawbacks such as cell size and power consumption at system level.^[^ ^9^, ^10^, ^11^, ^12^ ^]^ In order to address those issues, the novel technology advantages would be very appealing.

In recent years, the application of ferroelectric memories has attracted increasing attention,^[^ ^10^, ^13^, ^14^, ^15^ ^]^ However, the traditional ferroelectric memory including BiFeO_3_ and BaTiO_3_ exhibits poor properties for complementary metal‐oxide‐semiconductor (CMOS) fabrication. Until 2011, Böscke et al. reported ferroelectric hysteresis loops in silicon (Si)‐doped HfO_2_ thin‐film capacitors, which opens an avenue to fabricate CMOS compatible ferroelectric memories.^[^ ^16^ ^]^ Meanwhile, the ferroelectric behavior is also observed in the HfO_2_ thin films doped with aluminum (Al), zirconium (Zr), yttrium, gallium, lanthanum (La), and so on.^[^ ^17^, ^18^, ^19^, ^20^, ^21^, ^22^ ^]^ Among them, the Al‐doped HfO_2_ thin films show good thermal stability and low leakage current owing to the Al_2_O_3_ wide bandgap and strong Hf‐Al‐O bonding.^[^ ^23^, ^24^, ^25^ ^]^ However, the small remanent polarization hinders the development of Al‐doped HfO_2_,^[^ ^17^, ^26^, ^27^ ^]^ The doped element size has been a key parameter for the remanent polarization, and the large dopants seem generally favorable for large remanent polarization compared to others with a small ionic radius than hafnium.^[^ ^28^ ^]^ The trivalent dopants are generally a good choice to favor the polar phase in HfO_2_, which is confirmed theoretically.^[^ ^29^, ^30^ ^]^ The element of La is especially suitable to promote ferroelectricity.^[^ ^31^, ^32^ ^]^ The fabrication of vertical 3D ferroelectric memory was the first steps toward vertical for implementation of La and Al co‐doped HfO_2_ thin films (HfALAO). Such devices have several benefits over the conventional 3D NAND memory, including low power, high endurance and fast operations, while maintaining CMOS compatibility and high‐density.^[^ ^33^, ^34^, ^35^ ^]^

In this study, a vertical macaronic‐type 3D ferroelectric memory with ten transistors in series is reported. Meanwhile, the endurance characteristics are 10^12^ cycles. Under 85 °C, the endurance properties reach the 10^8^ cycles, which is the first demonstration for such devices.

## 2 Results and Discussion

**Figure 1:**
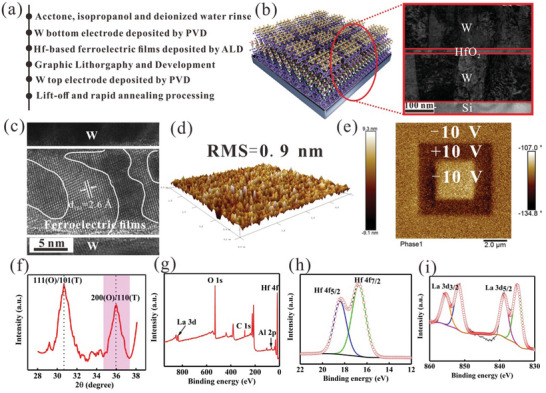
(a) the fabrication flow of W/HfO_2_‐based ferroelectric films/W Si devices, (b) schematic structure and the cross‐sectional images of W/ HfAlAO /W Si devices. (c,d) The TEM and AFM image of the HfALAO thin films, respectively. (e) PFM phase for the HfALAO thin films with −10 and +10 V. (f) GIXRD pattern of the HfALAO thin films. (g) XPS survey profile of the initial form of HfALAO and core‐level spectra assigned to (h) Hf 4f (i) La 3d, respectively.

We designed tungsten (W)/HfO_2_‐based ferroelectric films/W/Si devices, and **Figure** **1**a shows the fabrication steps. The structure of the HfALAO ferroelectric devices are shown in Figure 1b. The transmission electron microscopy (TEM) image shows the device cross‐section, where the boundaries between the layers are clear. Figure 1c shows that the HfALAO thin films are crystallized well through RTP process, and multiple phases co‐exist in the HfALAO thin films. The chemical maps reveal the distributions of W, Hf, O inside the HfALAO devices (see the Figure S2, Supporting Information). The d‐spacing values are 2.6 Å, which matched well with the X‐ray Diffraction (XRD) results (see the Figure 1f). The fast Fourier‐transform further revealed the crystal structure of the HALAO thin films (see the Figure S3, Supporting Information). Figure 1d shows that the surface topography of the HfALAO is the 0.9 nm root‐mean‐square, which exhibits good uniformity. Figure S4 (Supporting Information) shows that the surface topography of the HfALAO with 4.0% Al and 1.9% La and HfAlO thin films are 0.91 and 1.0 nm, respectively. As shown in Figure 1e, the outer and inner regions are observed at −10 and +10 V, and the bright and dark regions indicated upward and downward ferroelectric polarization. The piezo‐force microscopy (PFM) response is equal for +P and −P poled regions with ≈28° phase change, which proves the ferroelectricity of Hf‐based thin films. The ferroelectric domain can be observed in the HfALAO thin films. The domain size can reach 53.3 nm, as shown in Figure S5 (Supporting Information). The others can also gain similar phenomena and grain size. Figure 1f shows the XRD patterns of HfALAO thin films. The diffraction peaks are ≈30.5° and 35.9° referring to 111(O) and 200 (O) phase, which represent a high o‐phase fraction in HfALAO thin films. Figure 1g shows the XPS profiles of the HfALAO thin films (Hf (23.78%), La (4.20%), Al (2.17%) and O) with Hf/La/Al cycle ratio of 94:4:1. The HALAO (4.0% Al and 1.9% La) and Al doped hafnium‐based thin films (HfAlO thin films) also represent a high o‐phase fraction (see the Figure S6, Supporting Information). Figure 1h shows the Hf 4f with two peaks at ≈16.9 and ≈18.5 eV, which corresponded to Hf 4f_7/2_ and Hf4f_5/2_. The binding energies of La 3d_3/2_ are located at 855.6 and 851.5 eV, and those of La 3d5/2 are located at 838.8 and 834.9 eV (see Figure 1i). Figure S7a (Supporting Information) shows the characteristic peak located at ≈74.4 eV corresponding to Al 2p. The O 1s spectra with four chemical compositions are shown in Figure S7b (Supporting Information). The main peaks at 531.1. 530 and 529.1 eV are attributed to the Al‐O, Hf‐O, and La─O bonding peaks, respectively. The other peaks corresponded to the existence of the oxygen vacancies. The binding energy of Hf 4f and La 3d are approximately consistent with previous studies.

**Figure 2:**
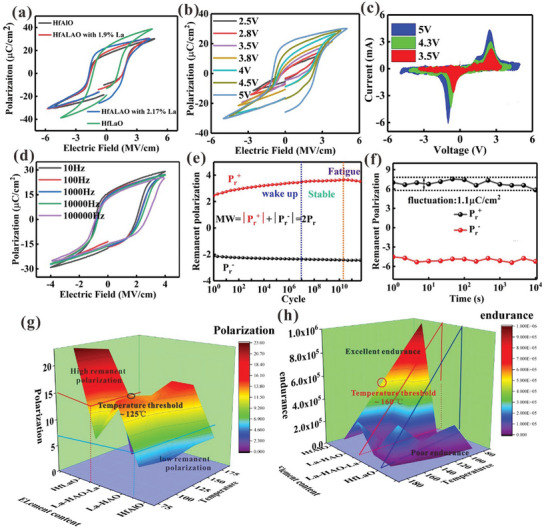
a) P‐E curves of the HfALAO devices with different Al and La content, P‐E curves of HfALAO thin films of 4.2% Al and 2.17% La b) P‐E curves of device under different voltages c) The HfALAO‐based ferroelectric *I–V* curve of the samples. d) P‐E curves of device under different frequent. e,f) fatigue properties and retention characteristics of the devices’ remanent polarization values, respectively. g) The effect of element content as well as temperature on the remanent polarization. h) The impact of the element content and temperature on the device endurance.

As shown in **Figure** **2**a, the HfALAO thin films (the Hf (23.78%), La (4.20%) and Al (2.17%)) show the saturated ferroelectric responses with the high remanent polarization of ≈22µC cm^−2^. With the decrease of the La content, the coercive fields are also changed. The Al‐doped HfO_2_ display the minimal coercive field of ≈1.6 V. Figure 2b shows the P‐E hysteresis curve of the HfALAO thin films (the Hf (23.78%), La (4.20%), and Al (2.17%)) at different voltage sweeping ranges. With increase in voltage sweep, larger hysteresis was observed. As shown in Figure 2c, the *I–V* curves show similar trend, which laid the foundation for good storage features. On the contrary, the P‐E curves of the devices deteriorated with increase in the frequency, as shown in Figure 2d. Furthermore, the fatigue characteristics of the devices are tested with the write voltage of 2 V and read voltage of 3.5 V. We can see that the HfALAO devices show excellent endurance of over 10^11^ cycles. The device failure process is divided into three stages including wake up, stable and fatigue process (see the Figure 2e). Meanwhile, the devices exhibits long retention properties up to 10^4^ s, but the device performance shows slight fluctuations with a remanent polarization difference of ≈1.1 µC cm^−2^, as shown in Figure 2f. Figures S8 and S9 (Supporting Information) show that the HALAO (4.0% Al and 1.9% La) and HfAlO thin film also display excellent ferroelectric characteristics. Meanwhile, the HfAlO thin films show robust retention characteristics, as shown in Figure S8e (Supporting Information). Figure S10 (Supporting Information) shows the test waveform diagram of fatigue test and tolerance test. Table S1 (Supporting Information) shows that our work displays excellent ferroelectric properties, which lay the foundation of high‐density integration of hafnium‐based electronic devices.^[^ ^36^, ^37^, ^38^, ^39^, ^40^ ^]^ Furthermore, we can test the memory properties of the HALAO thin films. Figure S11 (Supporting Information) shows that the Hf‐based ferroelectric memory devices represent the excellent storage properties. Thus, the devices are promising candidates for further non‐volatile memory applications. Figure 2g shows that the effect of the element content and temperature on the remanent polarization. For the la doped Hafnium‐based thin films (HfLaO thin films, the device polarization would show obvious degradation with the increasing of the test temperature (see the Figure S12, Supporting Information). When the test temperature is over 125 °C, the devices display failure. For the hafnium‐based thin films with aluminum element, the remanent polarization shows slight fluctuation with the increase of the test temperature. Furthermore, we explore the impact of test temperature and element content on the device endurance. When the hafnium‐based films contain aluminum elements, Figures S13 and S14 (Supporting Information) shows that the thermal endurance characteristics can be enhanced. As shown in Figure 2h, the performance of the ferroelectric devices is divided into three periods including excellent, normal and poor zone. For the HfLaO thin films, the devices show poor thermal stability. The HfAlO thin films show excellent thermal stability (see the Figure S15, Supporting Information). With the increase of the test temperature, the endurance cycles present obvious decline. When the test temperature is over the 160 °C, the endurance cycles of the hafnium‐based devices down to the ≈5×10^5^ under applied voltage of 3 V. The reason may be that the defect restricts the domain flipping (see the Figure S16, Supporting Information) Meanwhile, our devices show excellent mechanical flexibility, which lays the foundation of high‐density integration of flexible electronic devices (see the Figure S17, Supporting Information).

**Figure 3:**
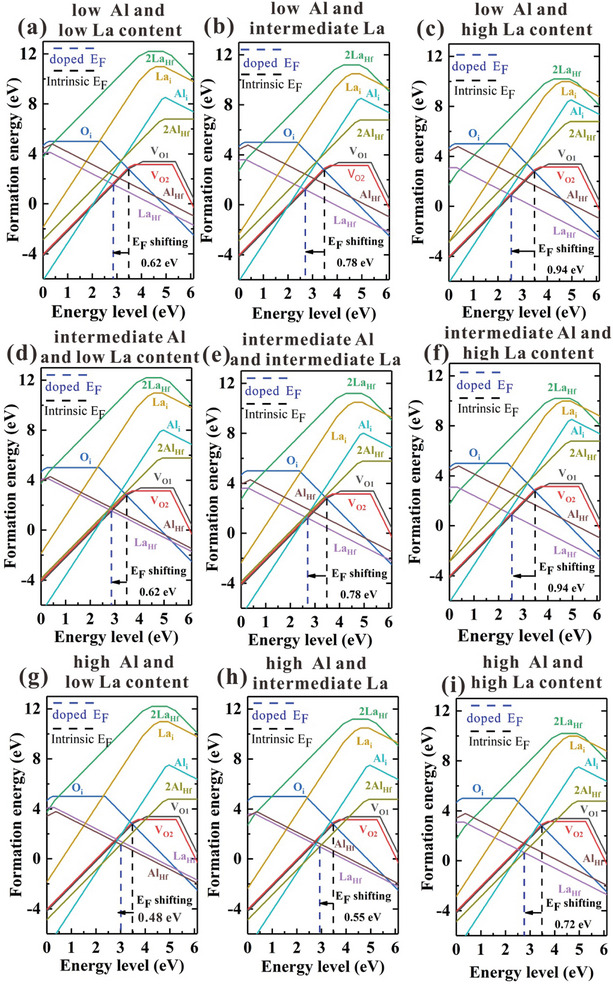
The variation of defect formation energies with Fermi level at low, intermediate and high Al content and low, intermediate and high La content. The major defects include intrinsic oxygen‐related defects O_i_ (interstitial oxygen), V_O1_ (3‐coordination oxygen vacancy), V_O2_ (4‐coordination oxygen vacancy); Al‐related defects, Al_Hf_ (one aluminum atom replacing one hafnium), 2Al_Hf_ (two aluminum atoms replacing one hafnium), Al_i_ (interstitial aluminum); and La‐related defects, La_Hf_ (one lanthanum atom replacing one hafnium), 2La_Hf_ (two lanthanum atoms replacing one hafnium), and La_i_ (interstitial lanthanum).

In theory, we calculated the effect of dopants on the ferroelectricity of HfALAO thin films. Our previous work has demonstrated that Al dopants play a significant role in controlling the density of oxygen vacancies and the o‐phase fraction, and the intermediate Al content is the optimum growth condition in fabricating HfAlO thin films with robust ferroelectricity.^[^ ^27^ ^]^ In this work, we further considered the effect of La and Al dopants on the ferroelectricity of HfALAO thin films using the same method. **Figure** **3** shows the formation energy of both intrinsic defects, Al‐related defects and La‐related defects varied with the Fermi level. Figure S18 (Supporting Information) shows that the structure of orthorhombic HfO_2_ without defect and with La‐related and Al‐related defect.

Influenced by the Al and La dopants, significant Fermi level shifting is observed in Figure 3, which is determined by the electrically neutral condition and can be qualitatively determined by the positively and negatively charged defects with the lowest formation energy. With no Al or La dopant considered, the Fermi level is dominated by the positively charged point defect $V_{O}^{2+}$ and negatively charged point defect $O_{i}^{2-}$ at 3.48 eV, as shown by the black dashed line in Figure 3a–i. Under low Al and intermediated Al condition, which can be seen in Figure 3a–f, when both the Al and La dopants are introduced, the ${\mathrm{La}}_{\mathrm{Hf}\;}^{-}$ defect becomes the main negatively charged defect since it has lower formation energy than $O_{i}^{2-}$ and ${\mathrm{Al}}_{\mathrm{Hf}}^{-}$. In this case, La dopants have greater effect on the Fermi level compared with Al dopants, and as a result, the Fermi level is determined by $V_{O}^{2+}$ and ${\mathrm{La}}_{\mathrm{Hf}\;}^{-}$ and is fixed at 2.86 eV at low La content (shown by the blue dashed line in Figure 3a,d). Afterward, as the La content increases, the formation energy of ${\mathrm{La}}_{\mathrm{Hf}\;}^{-}$ decreases, and the Fermi level shifts to the valance band (VBM) constantly. At the intermediate and high La content, the Fermi level decreases to 2.70 eV (shown by the blue dashed line in Figure 3b,e) and 2.54 eV (shown by the blue dashed line in Figure 3c,f) respectively. To conclude, at low and intermediated Al content, the Fermi level decreases constantly with the addition of La dopants due to the low formation energy of ${\mathrm{La}}_{\mathrm{Hf}\;}^{-}$ defect.

In comparison, the position of Fermi level at high Al content is different from that under low and intermediate Al content. Since the content of Al dopants increases, the formation energies of all Al‐related defects plunge. At high Al and low La content, the dominant charged defects become $2{\mathrm{Al}}_{\mathrm{Hf}}^{2+}$ and ${\mathrm{Al}}_{\mathrm{Hf}\;}^{-}$. So the Fermi level is fixed at 3.0 eV (shown by the blue dashed line in Figure 3g), which is higher than that at lower Al content and the same low La content. Afterward, at intermediate and high La content with high Al content, the formation energy of ${\mathrm{La}}_{\mathrm{Hf}}^{-}$ drops with the increase of La content, and becomes lower than that of ${\mathrm{Al}}_{\mathrm{Hf}}^{-}$. As a result, the Fermi level becomes determined by ${\mathrm{La}}_{\mathrm{Hf}}^{-}$ and $2{\mathrm{Al}}_{\mathrm{Hf}}^{2+}$ and is fixed at 2.93  and 2.76 eV respectively (shown by the blue dashed line in Figure 3h,i). Like the result under high Al content and low La content condition, the Fermi levels are higher than those at lower Al content and the same La content, which is due to the impact of $2{\mathrm{Al}}_{\mathrm{Hf}}^{2+}$ defect. The formation energy of $2{\mathrm{Al}}_{\mathrm{Hf}}^{2+}$ defect is lower than that of $V_{O}^{2+}$ defect, so the intersection of positively charged defect and negatively charged defect will move toward the conduct band (CBM) and finally the position of Fermi level changed. In conclusion, in La and Al co‐doped system, at high Al content, the dominated charged defects are different from that at lower Al content, causing the Fermi level to move toward CBM, and this conclusion corresponds to the result in HfAlO.^[^ ^10^ ^]^

Comparing the effect of La and Al content, it is easy to conclude that with the La dopants increasing, the Fermi level moves toward VBM constantly(compare the figures in Figure 3 laterally), but with the Al dopants increasing, the Fermi level moves toward VBM at first and then moves toward CBM(compare the figures in Figure 3 vertically). This discrepancy mainly comes from the different formation energies of $2{\mathrm{Al}}_{\mathrm{Hf}}^{2+}$ and $2{\mathrm{La}}_{\mathrm{Hf}}^{2+}.$ With Al dopants considered, the dominant positively charged defect is $2{\mathrm{Al}}_{\mathrm{Hf}}^{2+}$ under high Al condition, which shifts the Fermi level to the conduct band (CBM). However, with La dopants considered, the $2{\mathrm{La}}_{\mathrm{Hf}}^{2+}$ defects have much higher formation energy (reach up to 11.20 eV compared with 5.78 eV for $2{\mathrm{Al}}_{\mathrm{Hf}}^{2+}$ at neutral state) and thus can hardly influence the Fermi level, so the Fermi level keeps shifting toward the VBM even at high La condition.

The shifted Fermi level(shown in Figure S19a, Supporting Information) caused by the Al and La dopants will affect the formation energy of oxygen vacancy, which is believed to promote the formation of ferroelectric o‐phase in the polycrystalline film.^[^ ^41^ ^]^ Since the oxygen vacancies are positively charged at the qualitatively determined Fermi level, their formation energy decrease when the Fermi level shifts to the VBM. When no Al or La dopant is considered, the formation energies of $V_{O1}^{2+}$ and $V_{O2}^{2+}$ are 2.77  and 2.91 eV complemented by the negatively charged $O_{i}^{2-}$. With Al and La dopants considered, the dominant defect changes and the Fermi level shifts to the VBM, so the formation energies of $V_{O1}^{2+}$ and $V_{O2}^{2+}$ decrease, as shown in Figure S19b,c (Supporting Information). The variation trend of $V_{O}^{2+}$ formation energy keeps consistent with that of Fermi level. With the La dopants increase, the formation energy of $V_{O}^{2+}$ plunge, and with the Al dopants increases, it drops in the beginning and then raises. At intermediate Al content, the formation energies of $V_{O1}^{2+}$ and $V_{O2}^{2+}$ drop to 1.50  and 1.64 eV respectively at low La content and then decrease constantly to 0.87  and 1.03 eV at high La content. The lower formation energy indicates that the defect is more easily to form, which favor the formation of o‐phase HfO_2_ and promote the polarization of film.^[^ ^41^ ^]^

**Figure 4:**
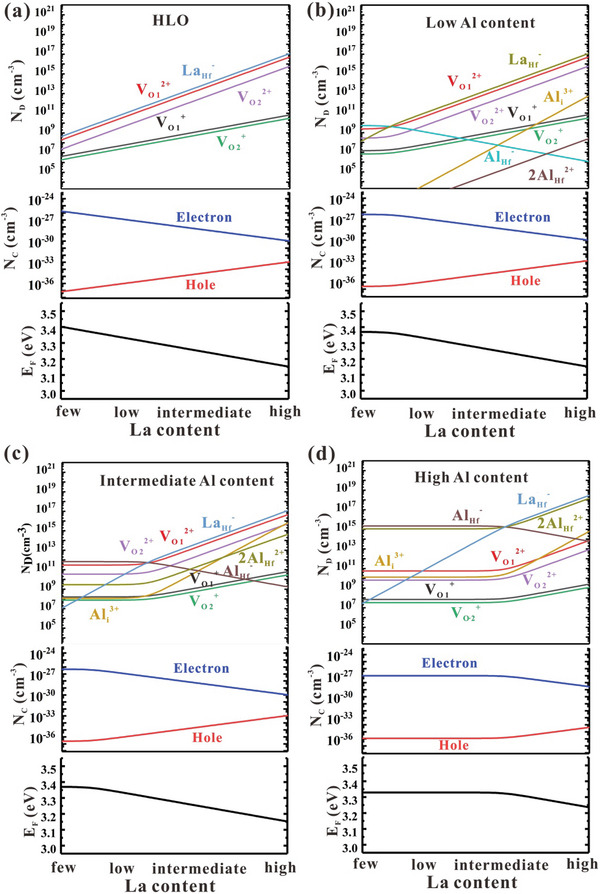
Variation of Fermi levels, carrier concentrations, and defect densities with La content under slightly O‐poor conditions a)without Al dopant, b) at low Al content, c) at intermediate Al content, and d) at high Al content.

In order to further quantitatively analyze the impact of doping on the defect formation, the Fermi levels, carrier concentrations, and defect densities varied with La content are calculated by the self‐consistent method^[^ ^42^ ^]^ under slightly O‐poor condition,^[^ ^41^ ^]^ as **Figure** **4** shows.

In the HfLaO film without Al doping, the $V_{O}^{2+}$ density exhibits an increasing trend with the increase of La content (see Figure 4a), which is inconsistence with the case in HfAlO with $V_{O}^{2+}$ density decreasing from intermediate Al content to high Al content.^[^ ^36^ ^]^ This discrepancy is mainly caused by the different density of positively charged dopant defects in HfAlO and HfLaO. In HfAlO, the $2{\mathrm{Al}}_{\mathrm{Hf}}^{2+}$ defects can massively form (reach 10^15^ cm^−3^ at high Al content) and cause the Fermi level to shift to the CBM with high Al content, as illustrated in Ref.[47] While in HfLaO, the densities of positively charged ${\mathrm{La}}_{i}^{3+}$ and $2{\mathrm{La}}_{\mathrm{Hf}}^{2+}$ defects are both very low even at high La content (lower than 10^3^ cm^−3^), so they can hardly affect the position of Fermi level and density of $V_{O}^{2+}$ defects. As a result, in the HfLaO film, the density of $V_{O}^{2+}$ increases constantly with La content from 10^7^  to 10^16^ cm^−3^ due to the ${\mathrm{La}}_{\mathrm{Hf}\;}^{-}$ defects, which is much wider than the range in HfAlO(from 10^7^  to 10^11^ cm^−3^), illustrating the significant modulation ability of La doping on $V_{O}^{2+}$ density. It has been experimentally proven that $V_{O}^{2+}$ defects favor the growth of large o‐phase grains and increase the proportion of o‐phase, so La doping can promote the polarization of thin films more effectively than Al doping.

Since it is suggested by Figures S12 and S13 (Supporting Information) that Al doping plays an important role in enhancing the endurance of the film, the films with better ferroelectric properties can be obtained by La and Al co‐doping. With both La and Al dopants considered, the densities of the $V_{O}^{2+}$ defects are determined by both negatively charged ${\mathrm{La}}_{\mathrm{Hf}\;}^{-}$ and ${\mathrm{Al}}_{\mathrm{Hf}\;}^{-}$ dopant defects under neutral condition, as Figure 4b–d shows. With relatively low La content, $V_{O}^{2+}$ density is dominated by ${\mathrm{Al}}_{\mathrm{Hf}\;}^{-}$ defect and is almost independent of La content, so it varies with different Al content. At low Al content, it remains at ≈10^9^ cm^−3^ from few to low La content. At intermediate Al content, it remains at ≈10^11^ cm^−3^ in a wider La content range. And at high Al content, it remains at ≈10^10^ cm^−3^ from few to intermediate La content because of the massive formation of positively charged competitor $2{\mathrm{Al}}_{\mathrm{Hf}}^{2+}$. Afterward, as La‐doping content increases, the ${\mathrm{La}}_{\mathrm{Hf}\;}^{-}$ density increases while the ${\mathrm{Al}}_{\mathrm{Hf}\;}^{-}$ density decreases accordingly. When the density of ${\mathrm{La}}_{\mathrm{Hf}\;}^{-}$ defects surpasses that of ${\mathrm{Al}}_{\mathrm{Hf}\;}^{-}$ defects, ${\mathrm{La}}_{\mathrm{Hf}\;}^{-}$ defects become the defect dominating the $V_{O}^{2+}$ density. With relatively high La content, the density of $V_{O}^{2+}$ defect increase accompanying with the increase of ${\mathrm{La}}_{\mathrm{Hf}\;}^{-}$ and finally reaches up to 10^16^ cm^−3^ at low and intermediate Al content under high La condition, which is similar to the case in HfLaO. While at high Al content, influenced by $2{\mathrm{Al}}_{\mathrm{Hf}}^{2+}$, the increase rate of $V_{O}^{2+}$ density declines, only reaches 10^13^ cm^−3^ under high La content. In summary, from the perspective of $V_{O}^{2+}$ density, in the La and Al co‐doped system, too much Al dopants will introduce $2{\mathrm{Al}}_{\mathrm{Hf}}^{2+}$ positively charged defects, which can compete with $V_{O}^{2+}$ and reduce the density of $V_{O}^{2+}$. While the introduction of La dopants does not have this consequence. Figure S20 (Supporting Information) shows the density of oxygen vacancy under different doping condition in La and Al co‐doped system.

Though La doping can increase the density of $V_{O}^{2+}$ defect which favors the formation of ferroelectric o‐phase, the density of ${\mathrm{La}}_{\mathrm{Hf}\;}^{-}$ defect can also be modulated to a quite high level, up to 10^17^ cm^−3^ under high La content as shown in Figure 4a–d. Since the radius of La atom is larger than that of Hf atom, the charged ${\mathrm{La}}_{\mathrm{Hf}\;}^{-}$ defects with high density will cause the lattice distortion amid the crystal structure, thus weakens the stability of ferroelectric phase and surpasses the polarization expression.^[^ ^10^ ^]^ Furthermore, as the La content increases in the co‐doped HfALAO system, the positively charged ${\mathrm{Al}}_{i}^{3+}$ and $2{\mathrm{Al}}_{\mathrm{Hf}}^{2+}$ defects will also increase with the negatively charged ${\mathrm{La}}_{\mathrm{Hf}\;}^{-}$ defects to satisfy the neutral condition. Since it has been demonstrated that $2{\mathrm{Al}}_{\mathrm{Hf}}^{2+}$ defect can harm the ferroelectricity of HfO_2_ by forming Al_2_O_3_ impurity,^[^ ^27^ ^]^ the content of La should not be too high from the perspective of raising the o‐phase ratio. Therefore, in the La and Al co‐doped system, the optimal Al content is intermediate content, and La dopants should also be controlled at appropriate content to balance the contradiction between increasing the $V_{O}^{2+}$ defect density to increase the polarization and reducing the Al_2_O_3_ impurity and avoiding introducing too much lattice distortions.

**Figure 5:**
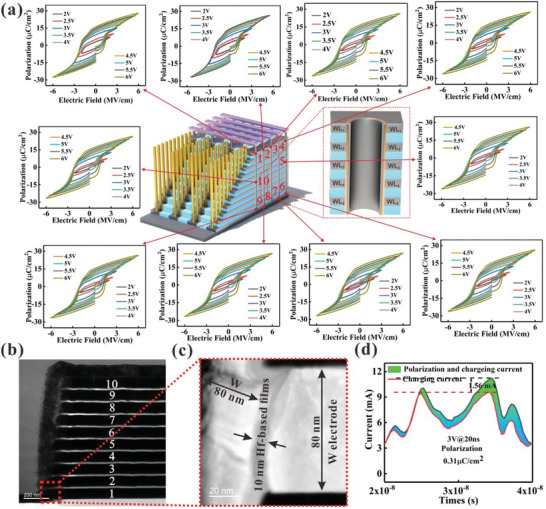
a) Schematic cross‐section of the macaroni‐type 3‐D ferroelectric devices with ten cells in series. b) Cross section of the 3D vertical structure with ferroelectric diode devices. c) The detailed structure information for the device, where a 80‐nm electrodes and 10‐nm hafnium‐based ferroelectric layers. d) The device response at voltage pulse of 20ns.

**Figure** **5**a shows the fabrication process of the 3D integration of hafnium‐based ferroelectric devices. Meanwhile, the 3D integration of hafnium‐based ferroelectric devices show excellent uniformity. Figure 5b shows the schematic view of the 10‐layer stacked ferroelectric devices. Figure 5c shows the cross‐section transmission electron microscopy (TEM) of the ten layer vertical structure, confirming the uniform HfO_2_ thin films. Figure S21 (Supporting Information) shows that the energy dispersive X‐ray spectroscopy (EDS) image of devices, confirming the uniform HfO_2_ thin films. Meanwhile, the 3D hafnium‐based device can be polarization under a test voltage of 20 ns, and the polarization can reach 0.31µC cm^−2^, as shown in Figure 5d. Furthermore, the hafnium‐based thin films display obvious synaptic properties including EPSC, PPF and LTP/LTD, as shown in Figure S22 (Supporting Information). Base on the LTP/LTD behavior of hafnium‐based devices, Figure S23 (Supporting Information) shows that the three‐layer arrays realize the recognition of handwritten digits. This lays the experimental foundation for the application of 3D hafnium‐based ferroelectric devices.

## 3 Conclusion

We investigated the influence of Al and La co‐doping HfO_2_ thin films on the ferroelectric characteristics of hafnium‐based devices. Among devices with different La/Al ratios, the HfAlO device with 4.2% Al and 2.17% La exhibited the excellent remanent polarization and thermostability, i.e., remanent polarization of 17 µC cm^−2^ and endurance times of 10^12^. Meanwhile, the ferroelectric memory still reach the 10^8^ cycles under 85 °C test temperature with write voltage amplitude of 2 V and frequency of 1 ms as well as read voltage amplitude of 3.5 V and frequency of 100 ns. Meanwhile, first principal analyses verified that hafnium‐based thin films with 4.2% Al and 2.17% La promoted the formation of the o‐phase against the paraelectric phase, providing theoretical support for supporting experimental results. A 3D ferroelectric memory is fabricated and shows excellent homogeneity.

## 4 Experimental Section

First, Si substrates were successively cleaned using acetone, absolute ethyl alcohol, and deionized water. Subsequently, the W electrodes were successively deposited on the Si substrates using physical vapor deposition (PVD). Then, the hafnium‐based thin films with thickness of ≈10 nm were deposited on the W/Si substrates using atomic layer deposition (ALD). Tetrakis‐(ethylmethylamino)‐hafnium (TEMAH) as well as trimethylaluminium (TMA) were used as precursors and were alternately vaporized into the ALD chamber by N2 carrier gas flow (TEMAH and TMA ratio = 34:1). H2O was used as an oxidant. Further, a 80 nm W metal layer was deposited by PVD, and RTP was performed in N2 atmosphere for 30 s at 550 °C.

Preparation process of the 10‐layer 3D vertical memory with ferroelectric memory cell is shown as follows.^[^ ^43^, ^44^ ^]^ The multiple W (80 nm)/Al_2_O_3_ (20 nm) layer are deposited by PVD and ALD, respectively. Patterning and etching were applied to form stacked wordlines (WL). Then, a 10‐µm hole is etched down to the bottom SiO_2_. Then, the Hf‐based thin films of ≈10 nm are deposited on the sidewall by ALD. The TEMAH, TMA and La‐FMD are used as Hf, Al and La precursors and alternately vaporized into the ALD chamber by the N_2_. The O_2_ plasma is used as the oxidant. The Al and La content of Hf‐based thin films is regulated by the ALD sub‐cycles of HfO_2_, Al_2_O_3_ and La_2_O_3_ at ALD chamber of 250 °C. Patterning and W are deposited as the pillar electrode (BL). The area of the memory cell is defined by the thickness of the bottom electrode W (80 nm) and the perimeter of the hole.

In order to explore how La and Al co‐doped affects the ferroelectricity of HfO_2_, the density functional theory (DFT) calculation were performed on the defect properties in HfALAO thin film.^[^ ^45^ ^]^ First, the formation energies of intrinsic, dopant Al‐related and dopant La‐related defects are calculated in a 2×2×2 supercell of orthorhombic HfO_2_ according to Equation (1).^[^ ^46^ ^]^

(1) $$\Delta E_{f}\left(\alpha ,q,E_{F}\right)=E^{\mathrm{total}}\left(\alpha ,q\right)-E_{\mathrm{host}}^{\mathrm{total}}+\sum n_{i}\mu_{i}+q\left(E_{\mathrm{VBM}}+E_{F}+\Delta V\right)$$

where ΔE_f_(α,q, E_F_) illustrates the formation energy of defect α with charge state q determined by the Fermi level E_F_, and E^total^(α,q) and $E_{\mathrm{host}}^{\mathrm{total}}$ are the total energy of defective structure and defect‐free structure respectively. µ_i_ is the chemical potential of each element, which is determined following the flowchart in Figure S1 (Supporting Information). The slightly O‐poor condition was selected to simulate the experimental growth environment.^[^ ^41^ ^]^

Then the density of defect α in charge state q can be derived from its formation energy and the self‐consistent Fermi level by the formula^[^ ^47^ ^]^:

(2) $$N\;\left(\alpha ,q,E_{F}\right)=N_{\mathrm{site}}\;g_{q}e^{-\Delta E_{f}\left(\alpha ,q,E_{F}\right)/k_{B}T}$$

In this work, the defect densities were calculated at an annealing temperature of 800 K and an operating temperature of 300 K. And the calculation is based on the electrically neutral condition considering all the defects. Throughout the calculation, the Heyd‐Scuseria‐Ernzerhof (HSE) hybrid function was used to get the correct bandgap and defect energy level with the projector augmented pseudopotentials.^[^ ^48^ ^]^ The energy cutoff was set to 520 eV and a single Γ‐point was used in the calculation of the 96‐atoms supercell.^[^ ^42^, ^49^ ^]^

## Conflict of Interest

The authors declare no conflict of interest.

## Supporting information



Supporting Information

## Data Availability

The data that support the findings of this study are available from the corresponding author upon reasonable request.
