# Supplementary material for: Effect of Lanthanum‐Aluminum Co‐Doping on Structure of Hafnium Oxide Ferroelectric Crystals
Source: Adv Sci (Weinh). 2024 Dec 4;12(4):2410765. doi: 10.1002/advs.202410765 (PMC11775560; doi:10.1002/advs.202410765)
Supplement: Supplementary file 1 — Supporting Information [file ADVS-12-2410765-s001.docx]

Effect of lanthanum-aluminum co-doping on structure of hafnium oxide ferroelectric crystals

The supporting information includes:

**S1 Flow chart for chemical potential determination**

**S2 Cross-sectional image of ferroelectric devices, and the line scan of Hf, Al, La, O,W, Si of the cross-sectional image**

**S3 the fast fourier transform pattern of HfAlAO thin films**

**S4 (a) the 2×2μm^2^ AFM images of device with the Hf/Al cycle of 34:1. (b) the 2×2μm^2^ AFM images of device with the Hf/Al/La cycle of**

**S5 PFM amplitudes and phase for the different content Al and La HALAO thin films.**

**S6 GIXRD pattern of the HfAlO thin films and the HALAO thin films with 4.0% Al and 1.9% La.**

**S7 core-level spectra assigned to Al 2p and O 1s.**

**S8 The ferroelectric characteristics of the HfAlO thin films**

**S9 The ferroelectric characteristics of the HALAO thin films with 4.0% Al and 1.9% La.**

**Table S1. Ferroelectric characteristics comparison of Hf-based ferroelectric devices.**

**S10 the test waveform diagram of fatigue test and tolerance test.**

**S11 Storage properties of HALAO thin films with different Al and La components**

**S12 the thermal endurance of HfLaO thin films under 85 and 125 ℃， respectively.**

**S13 the thermal endurance of HfAlAO thin films (4.0% Al and 1.9% La) under 85, 125, 150 and 175 ℃, respectively.**

**S14 the thermal endurance of HfAlAO thin films (2.17% Al and 4.2% La) under 85, 125, 150 and 175 ℃, respectively.**

**S15 the thermal endurance of HfAlO thin films under 85, 125, 150 and 175 ℃, respectively.**

**S16 The failure mechanism of film damages under high temperature.**

**S17 Hafnium-based ferroelectricity devices for flexible application**

**S18 The structure of orthorhombic HfO_2_ without defect and with La-related and Al-related defect.**

**S19 Fermi level and formation energy of oxygen vacancy under different doping condition in La and Al co-doped system.**

**S20 Density of oxygen vacancy under different doping condition in La and Al co-doped system**

**S21 EDS mapping of Hf, O and W**

**S22 Synaptic properties of hafnium-based thin films**

**S23 Image recognition of hafnium-based devices**

**S1 Flow chart for chemical potential determination**

**
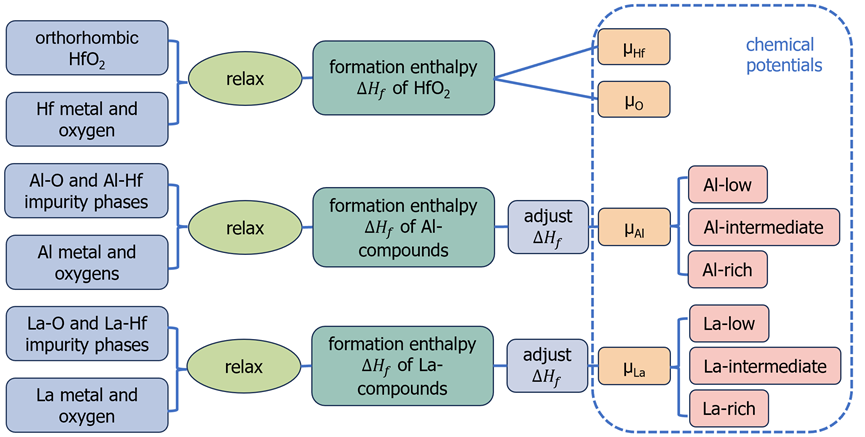
**

**Figure S1**: Flow chart for chemical potential determination.

To calculate the formation energy of intrinsic, dopant Al-related and dopant La-related defects according to Eq.1, the chemical potential of Hf, O, Al and La elements should be determined firstly following the steps in the flow chart below, as shown in Fig. 1S.

First, the structure of the simple substance and the target compound (such as HfO_2_, Al_2_O_3_) should be optimized, and then the formation enthalpy of the target compound should be calculated. Finally, the chemical potential of elements Hf and O can be determined according to the formation enthalpy of HfO_2_, and the chemical potential of Al and La at low, intermediate and high content can be modulated based on the formation enthalpy of related Al and La compounds.

**S2 Cross-sectional image of ferroelectric devices, and the line scan of Hf, Al, La, O,W, Si of the cross-sectional image**


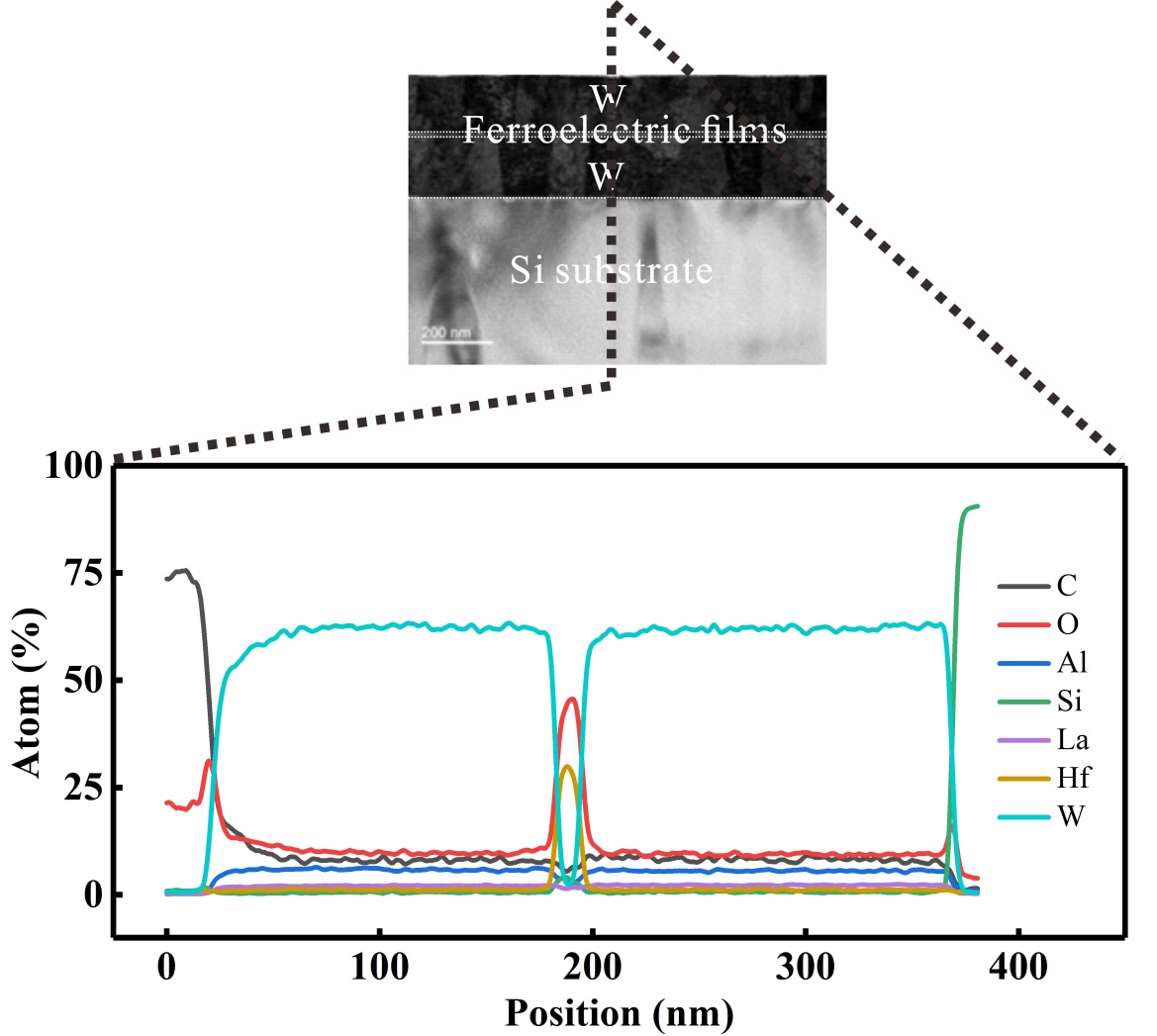


Fig. S2 cross-sectional image of W/HALAO/W/Si devices, and the line scan of Hf, Al, La, O, W and Si of the cross-sectional image

Fig. S2 the cross sectional image of the W/HfAlAO/W/Si ferroelectric device is clear. In order to obtain the element distribution, the line scan of the cross-sectional images is used as the measurement method. The constituent elements of Hf, O, W and Si are uniformly distributed in the films. The aluminum and aluminum are difficult to detect due to the element low content.

**S3 the fast fourier transform pattern of HfAlAO thin films**


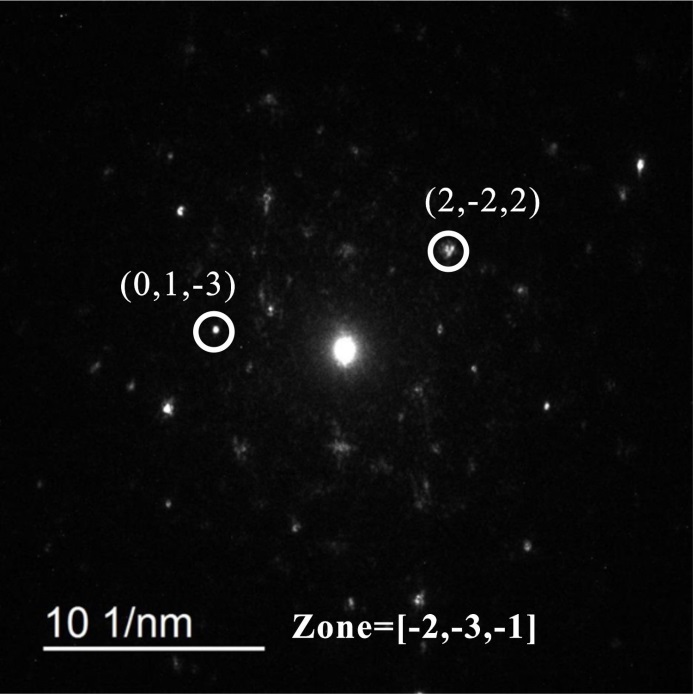


Fig. S3 the fast fourier transform pattern of HfAlAO thin films

Fig. S3 shows that the HfAlAO thin films crystallized well under 550 ℃.

**S4 (a) the 2×2μm^2^ AFM images of device with the Hf/Al cycle of 34:1. (b) the 2×2μm^2^ AFM images of device with the Hf/Al/La cycle of 49:1:1.**


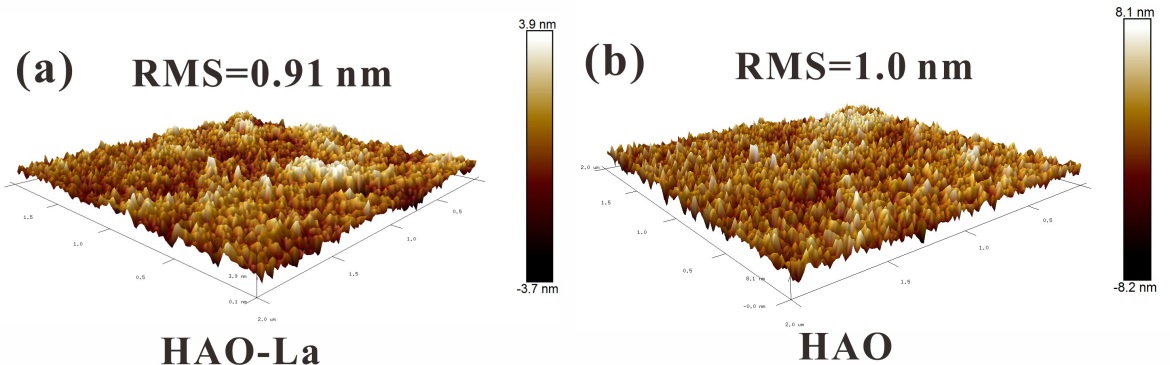


Fig. S4 the 2×2μm^2^ AFM images of device with the (a) Hf/Al cycle of 34:1 and (b) Hf/Al/La cycle of 49:1:1.

The surface morphology of Hf-based ferroelectric thin films is measured using AFM in the tapping mode. Fig. S4 (a) and (b) show the roughness is 0.91 and 1 nm for the HfAlO thin film with the Hf/Al cycle ratio of 34:1 and the HfAlAO thin films with the Hf/Al/La cycle ratio of 49:1:1. Those prove that our films have good uniformity.

**S5 PFM amplitudes and phase for the different content Al and La HALAO thin films.**


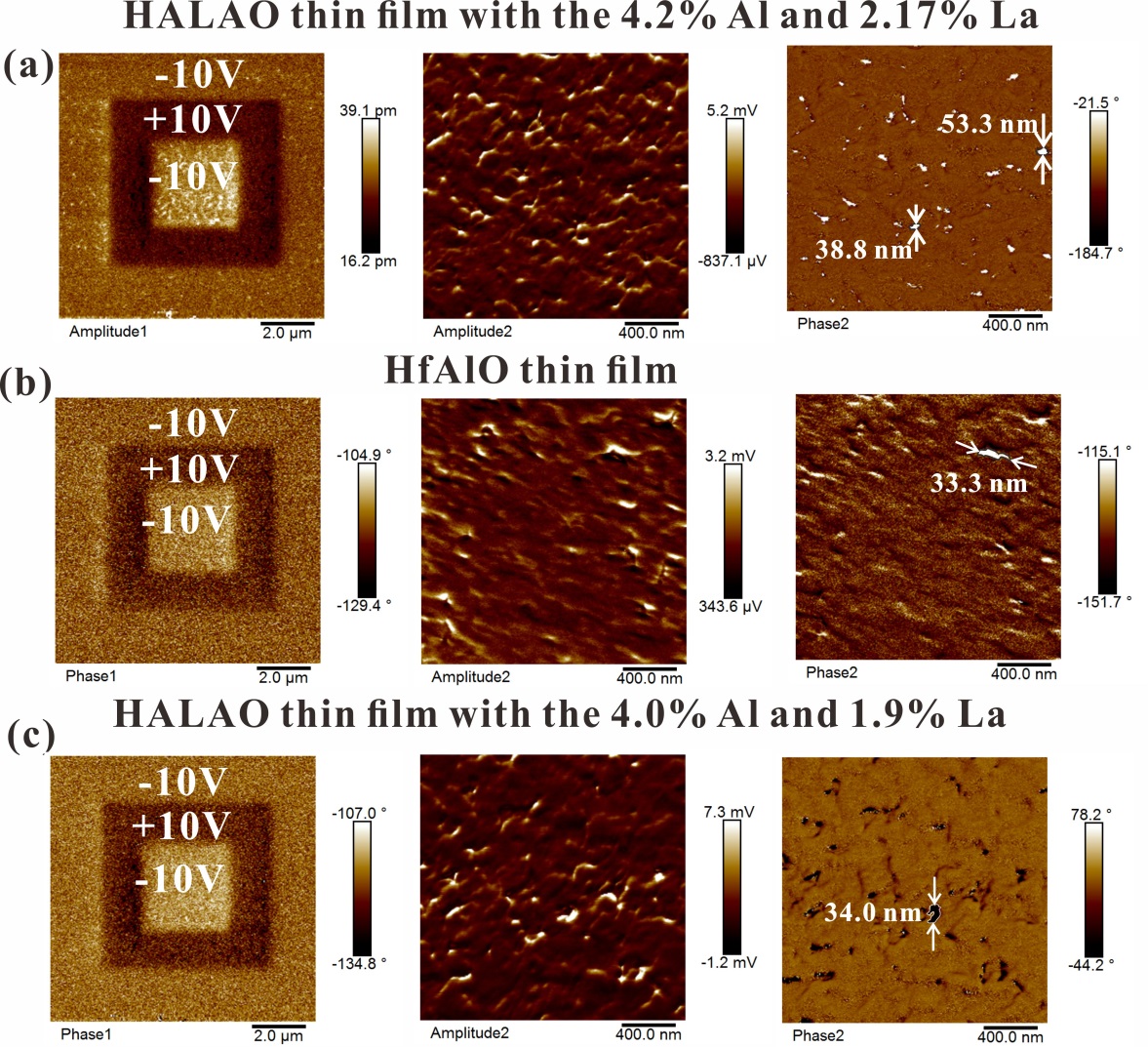


Fig. S5 PFM amplitude and phase for HALAO thin films with (a) 4.2% Al and 2.17% La, (b) HfAlO and (c) 4.0% Al and 1.9% La .

Strain distortion and polarization are believed to be coupled in HfO_2_ compounds. To verify the ferroelectricity of the HALAO thin films, large electrical field can be locally applied in PFM. PFM image illustrate uniform response, and nanoscaled ferroelectric domain can be observed with a driving voltage (2V AC), as shown in Fig. S5(a). To explore polarization dynamics, writing experiments are performed in which a square with lateral size of 10 μm is poled with -10 V bias followed by a 6 μm square using +10 V, and finally a smaller inner 3 μm square is again poled with -10 V bias. The contrast of out-of-plane PFM images are presented in Fig. S5 (b) and (c), which correlated with the orientation of the ferroelectric polarization, with the bright (dark) region indicating upward (downward) ferroelectric polarization. PFM image illustrate uniform response, and nanoscaled ferroelectric domain of 38.8, 33.3 and 34.0 nm can be observed in Fig. S5 (a), (b) and (c), respectively.

**S6 GIXRD pattern of the HfAlO thin films and the HALAO thin films with 4.0% Al and 1.9% La.**

Fig. S6 GIXRD pattern of the (a) HfAlO thin films and (b) the HALAO thin films with 4.0% Al and 1.9% La.

Fig. S6 (a) and (b) shows the XRD patterns of the HfAlO and HALAO thin films. The diffraction peaks of the HfAlO thin films are about 30°and 34°referring to 111(O) and 200 (O) phases. Similarly, Fig. S6 (b) shows that the diffraction peaks of the HALAO with 4.0% Al and 1.9% La are ~31.5°and 36°referring to 111(O) and 200 (O) phases. Those represent a high o-phase fraction in the HALAO thin films with different

**S7 core-level spectra assigned to Al 2p and O 1s.**

**
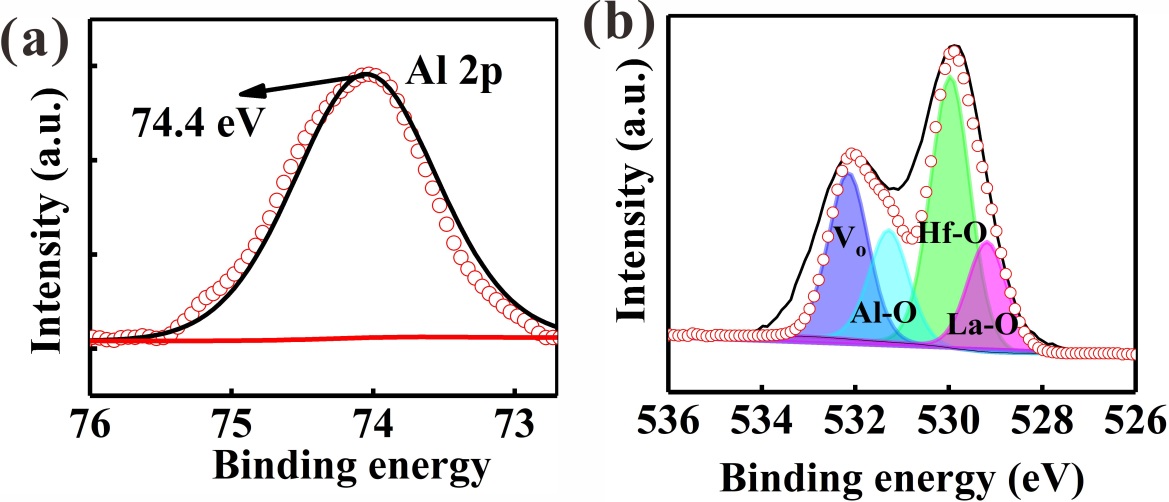
**

Fig. S7 core-level spectra assigned to (a) Al 2p and (b) O 1s.

Figure. S7 (a) shows the characteristic peak located at about 74.4 eV corresponding to Al 2p, which comprised Al^3+^. The O 1s spectra with four chemical compositions are shown in Figure. S7 (b). The main peaks at 531.1. 530 and 529.1 eV are attributed to the Al-O, Hf-O and La-O bonding peaks, respectively. The other peaks corresponded to the existence of the oxygen vacancies.

**S8 The ferroelectric characteristics of the HfAlO thin films**


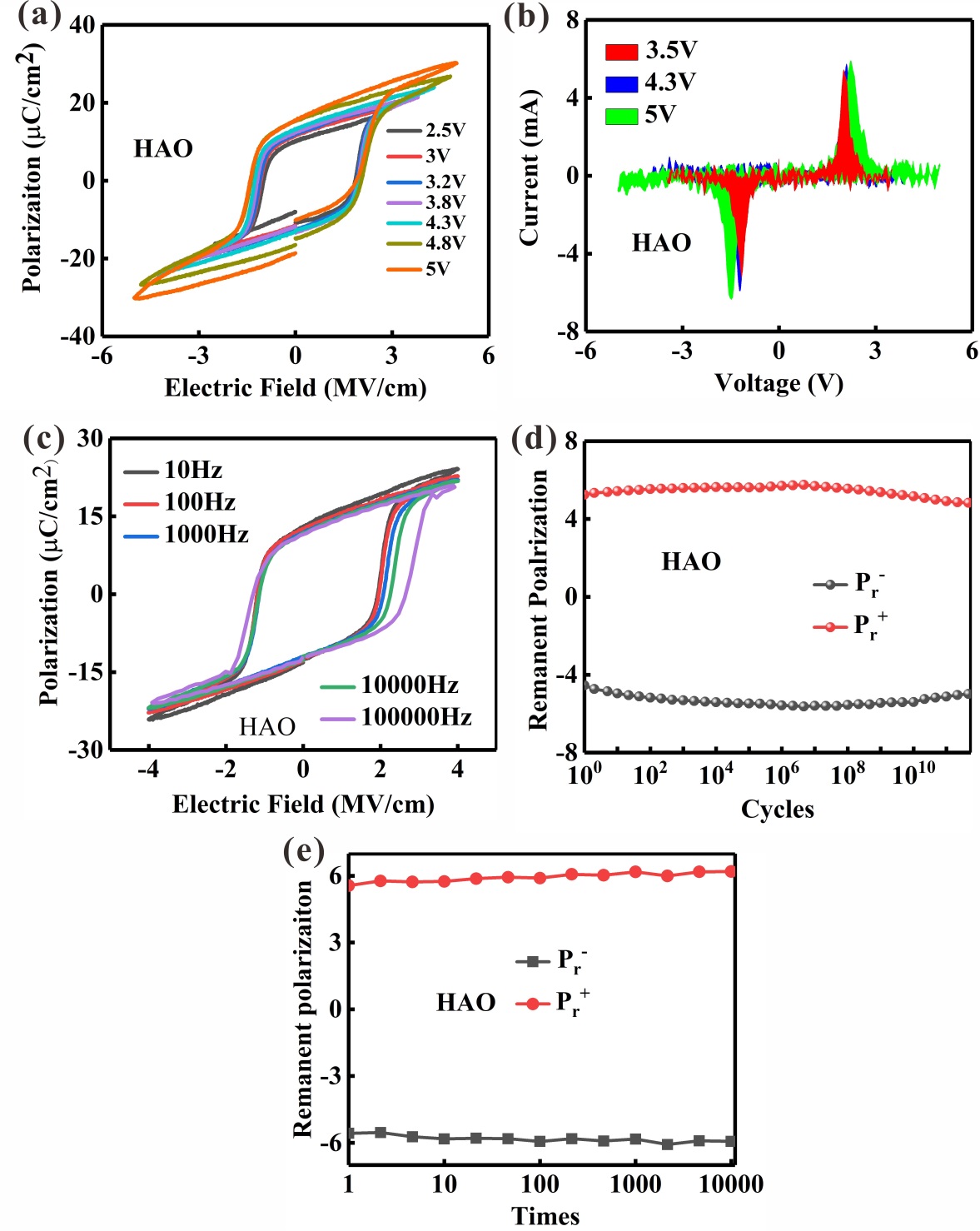


Fig. S8 (a) P-E hysteresis loop and (b) I-V curves of the HfAlO thin films under different sweeping voltages. (c) P-E hysteresis loop of the HfAlO thin films under different frequency. (d) and (e) fatigue properties and retention characteristics of the devices’ remanent polarization values, respectively.

Figure. S8 (a) shows the P-E hysteresis curve of the HfAlO thin films at different voltage sweeping ranges. With increase in voltage sweep, larger hysteresis was observed. Similarly, the I-V curves peak increase with the increase of the sweeping voltages, as shown in Fig. S8 (b). On the contrary, the P-E curves of the devices deteriorated with increase in the frequency, as shown in Fig. S8(c). Furthermore, the fatigue characteristics of the devices are tested with the write voltage of 2 V and read voltage of 3.5 V. We can see that the HfAlO devices show excellent endurance of over 10^11^ cycles (see the Fig. S8(d)). Meanwhile, the devices exhibits long retention properties up to 10^4^ s, as shown in Fig. S8 (e). Those laid the foundation for good storage features.

**S9 The ferroelectric characteristics of the HALAO thin films with 4.0% Al and 1.9% La.**


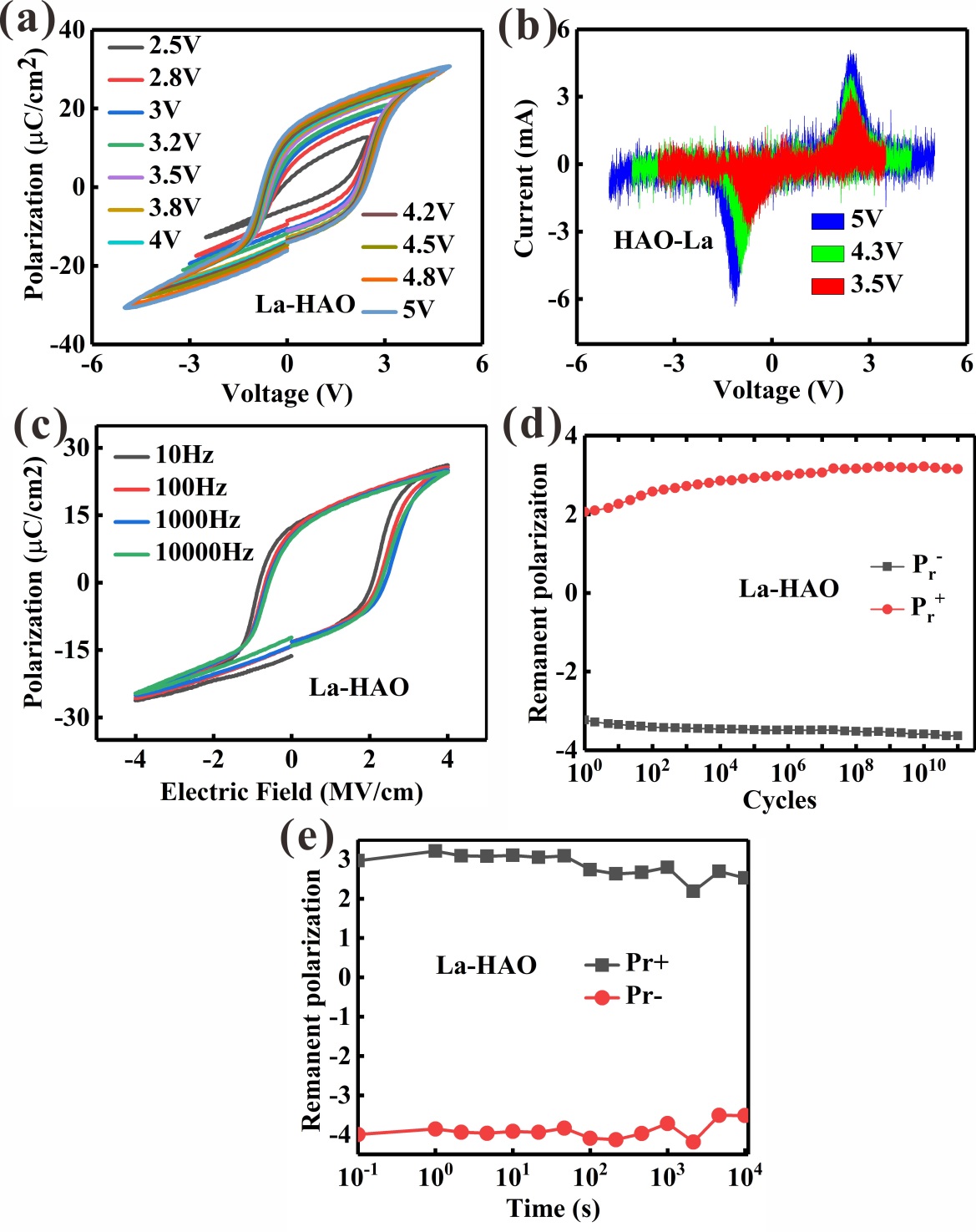


Fig. S9 (a) P-E hysteresis loop and (b) I-V curves of the HALAO thin films with 4.0% Al and 1.9% La under different sweeping voltages. (c) P-E hysteresis loop of the HfAlO thin films under different frequency. (d) and (e) fatigue properties and retention characteristics of the devices’ remanent polarization values, respectively.

For the HALAO thin films with 4.0% Al and 1.9% La, the P-E hysteresis loop and I-V curves under different sweeping voltages are similar pattern with the HfAlO thin film, as shown in Fig. S9 (a) and (b). Under the different sweeping frequent, the P-E hysteresis loop also show similar trend (see the Fig. S9 (c)). However, the P-E curve shows serious deformation under the over 100KHZ frequent. Furthermore, the fatigue characteristics of the devices are tested with the write voltage of 2 V and read voltage of 3.5 V. We can see that the HfAlO devices show excellent endurance of over 10^11^ cycles (see the Fig. S9(d)). Meanwhile, the devices exhibits long retention properties up to 10^4^ s, but the device performance shows obvious fluctuations, as shown in Fig. S9 (e).

**Table S1. Ferroelectric characteristics comparison of Hf-based ferroelectric devices.**

| Ferroelectric material | HfLaO | HfAlO | HZO | HfAlO | HfAlO | La-doped HfAlO |
| --- | --- | --- | --- | --- | --- | --- |
| Top electrode | W/TiN | Au/Ti | TiN | TiN/Ti | TiN | W |
| Bottom electrode | TiN | Pt | TiN | Si | TiN | W |
| Substrate | Silicon | Mica | Silicon | Silicon | SiO_2_/Si | SiO_2_/Si |
| Deposition technique | ALD | ALD | ALD | ALD | ALD | ALD |
| thickness (nm) | 10 | 10 | 12 | 10 | 10 | 10 |
| Pr (μC/cm^2^) | 20 | ~13 | ~20 | ~22 | ~18 | ~22 |
| Ec(MV/cm) | 1.6 | 1.5 | 2 | 3.6 | 1.2 | 1.5 |
| Endurance (cycles) | 10^6^ | >10^5^ | 10^9^ | 10^9^ | 10^8^ | Over 10^10^ |
| Reference | [47] | [48] | [49] | [50] | [51] | This work |

Comparisons of some basic parameters are made between this work and other reports, as shown in Table S1. In comparison with other Hf-based ferroelectric devices, our work shows excellent ferroelectric characteristics, which lay the foundation of high-density integration of hafnium-based electronic devices.

**S10 the test waveform diagram of fatigue test and tolerance test.**


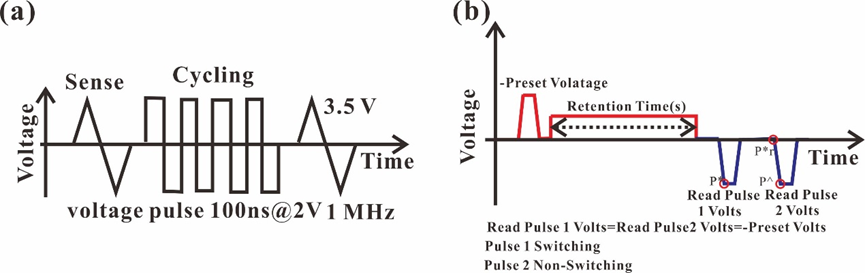


Fig. S10 the test waveform diagram of (a) fatigue test and (b) tolerance test.

For the fatigue test, Fig. S10(a) shows that the write voltage is the amplitude of 2V and the pulse of 100 ns. The read voltage is the amplitude of 3.5V and the pulse of 1000ns. For the tolerance test, Fig. S10(b) shows that the test voltage is 3V. The read voltage is the same of that of fatigue test.

**S11 Storage properties of HALAO thin films with different Al and La components**


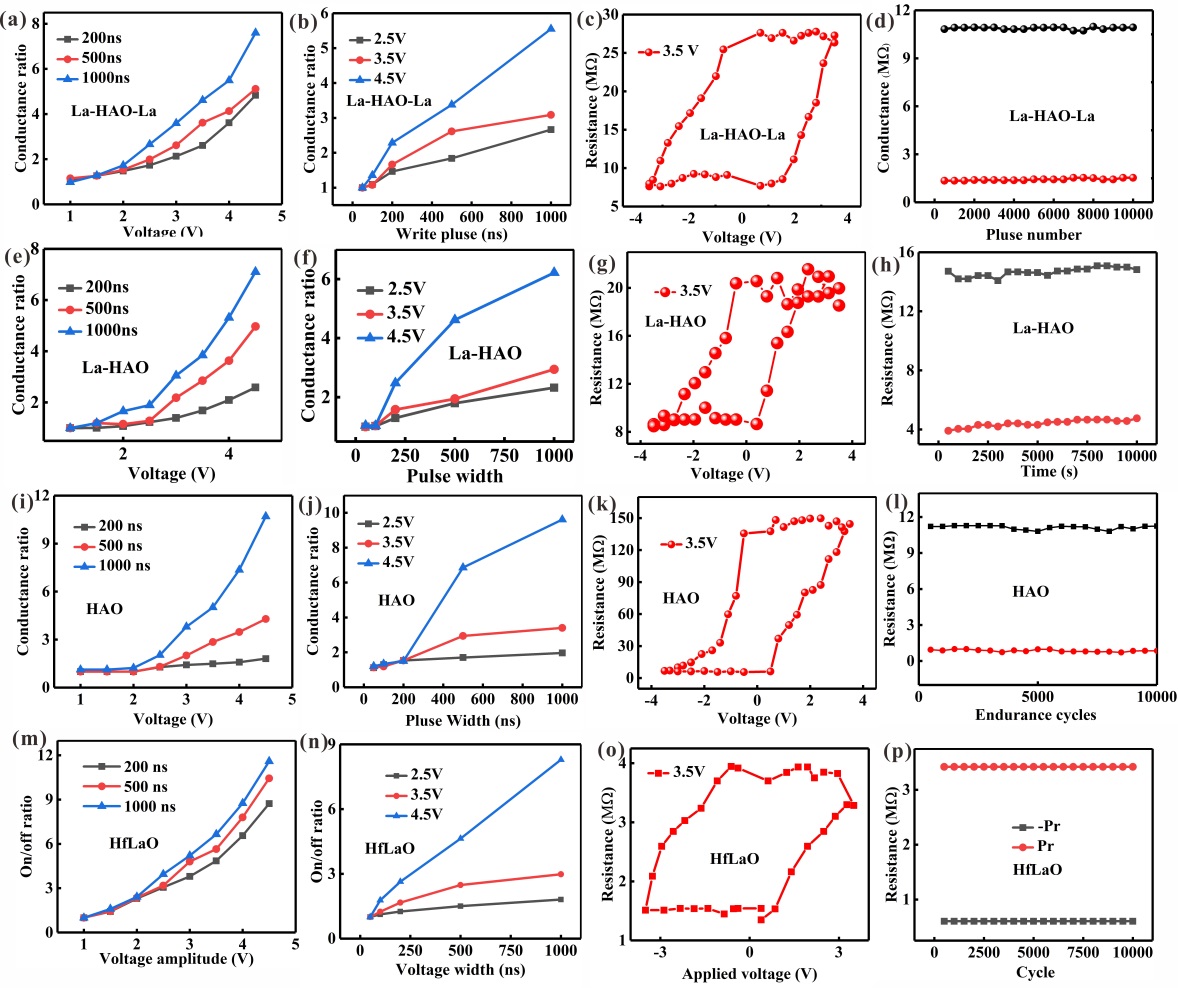


Fig. S11(a), (e), (i) and (m) dependence of conductance ratio on write voltage under various pulse width, (b), (f) , (j) and (n) change of conductance ratio with pulse width, (c), (g), (k) and (o) electrical resistance switching loop of HfAlO FTJs, (d), (h), (l) and (p) endurance properties of HALAO thin films with 4.2% Al and 2.17% La, HALAO thin films with 4.0% Al and 1.9% La, HfAlO thin films and HfLaO thin films, respectively.

We tested the storage performance of the HALAO thin films.When the pulse width reaches 1000 nm, the largest on/off conductance of ~ 8 is observed, as shown in Fig. S11(a). Similarly, with increase in pulse width, Fig. S11(b) displays that the conductance ratio also increases. For the write voltages of 4.5V, the conduction ratio is at maximum of about 6. Furthermore, the non-volatile resistance switching (R-V) of the samples was tested with pulse sequences of 0.5 V steps and different voltage amplitudes (see the Fig. S11(c)). Firstly, the positive pulse is applied to the top electrode, i.e., off-state. Then, the inverse pulse with of 1000ns width is applied to obtain the R-V loop. To understand the fatigue characteristics of the HALAO thin films, the devices are tested at sweeping amplitude of 4 V with pulse sequences of 0.5V steps and pulse width of 500 ns. Fig. S11(d) shows that HfAlO FTJs performance is almost unaffected when the pulse times reach the 10^4^. The other devices show similar trend. Meanwhile, the HfALO thin film show the largest on/off ratio of ~ 12 under voltage amplitude of 4.5 V and voltage width of 1000 ns.

**S12 the thermal endurance of HfLaO thin films under 85 and 125 ℃， respectively.**


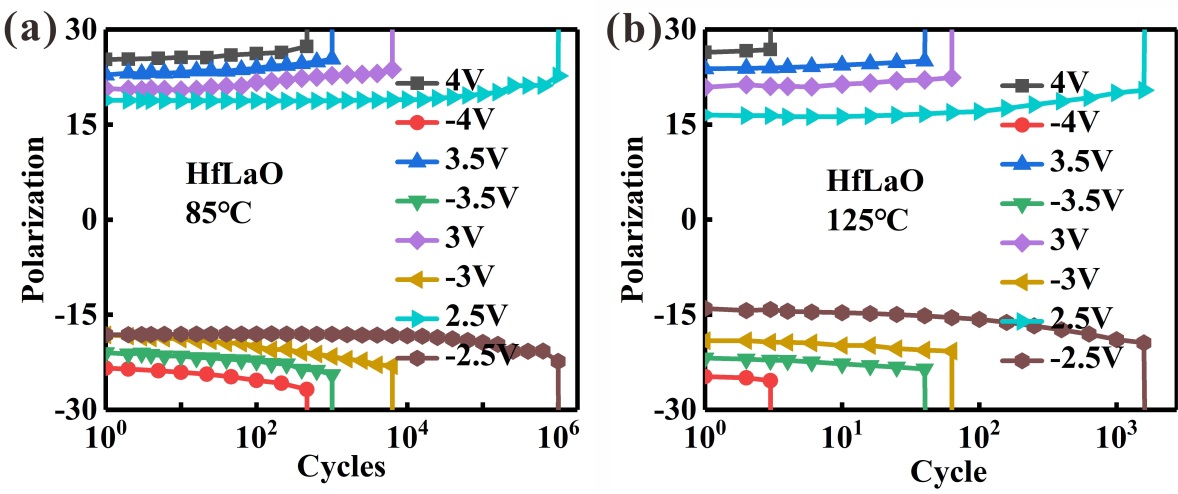


Fig. S12 (a) and (b) the endurance characteristics of the HfLaO thin films at 85 and 125 ℃，respectively.

Furthermore, we test the thermal endurance of the HfLaO thin films. As shown in Fig. S12(a), the endurance cycles show obvious degradation with the increase of applied voltage under 85 ℃. With the increase of the test temperature, the endurance display obviously degradation under 125 ℃ (see the Fig. S12(b)).

**S13 the thermal endurance of HfAlAO thin films (4.0% Al and 1.9% La) under 85, 125, 150 and 175 ℃, respectively.**


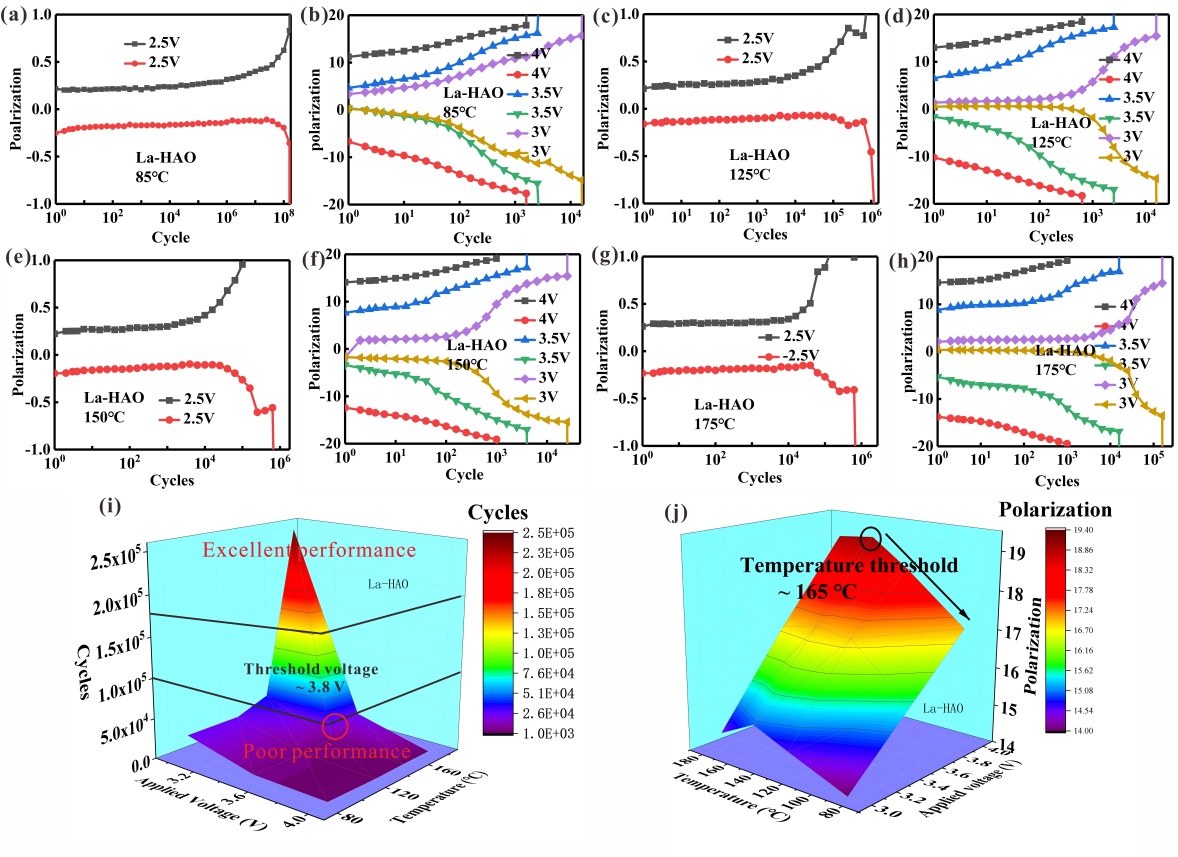


Fig. S13 (a), (b), (c), (d), (e), (f), (g) and (h) the endurance characteristics of the HfAlAO thin films (4.0% Al and 1.9% La) under 85, 125, 150 and 175℃, respectively. (i) The effect of applied voltages and temperature on the device endurance. (j) The impact of temperature and cycles on the device polarization.

As shown in Fig. S13(a)-(h), the HfAlAO shows similar trend with the HfLaO thin films with the increase of the applied voltage. However, the HfAlAO thin films show excellent thermal endurance. When the test temperature is at 175 ℃，the endurance cycles can reach 10^6^ under applied voltages of 2.5 V. Fig. S13(i) shows the endurance cycles shows rapidly descend over applied voltages of ~3.4V. As shown in Fig. S13(j), the polarization of HfAlAO thin films is minimum of about 19 μC/cm^2^ at test temperature of about 165 ℃, which may be caused due to the formation of oxygen defect.

**S14 the thermal endurance of HfAlAO thin films (2.17% Al and 4.2% La) under 85, 125, 150 and 175 ℃, respectively.**


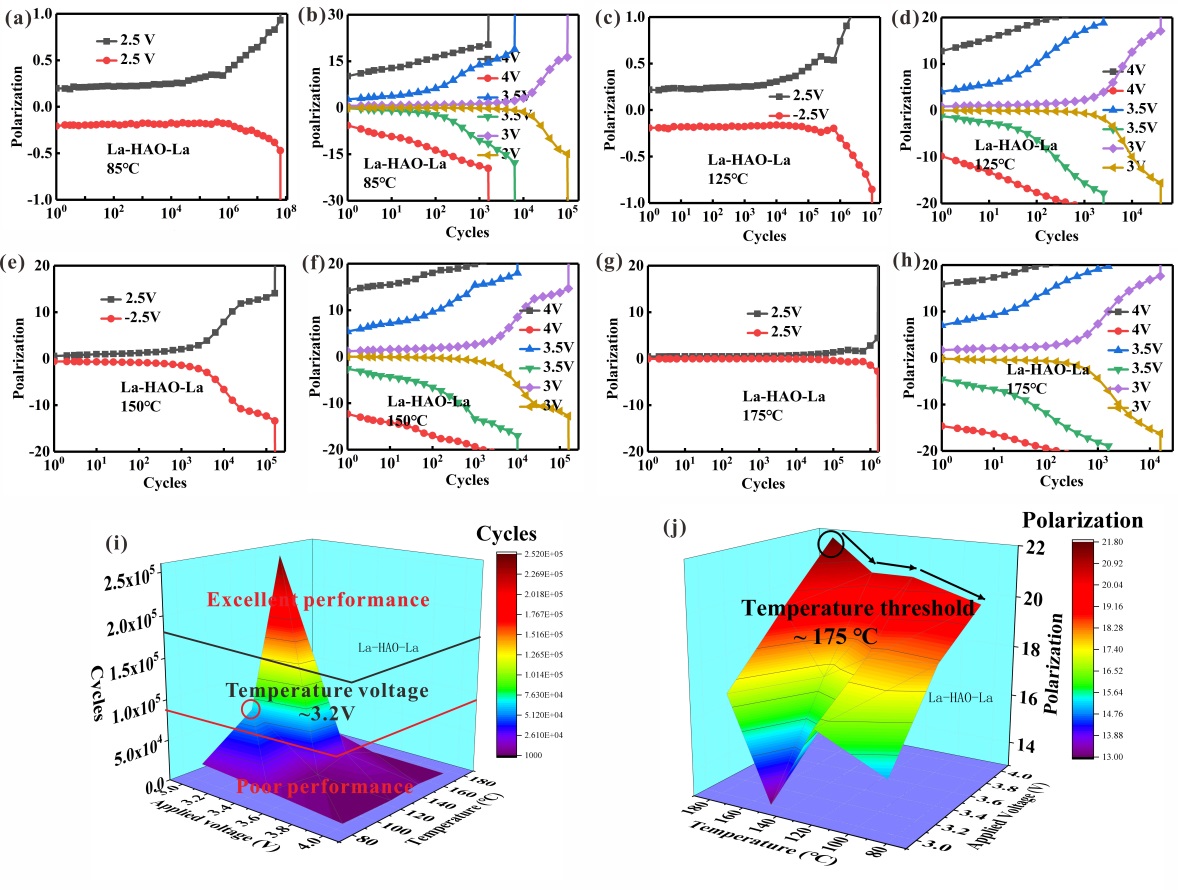


Fig. S14 (a), (b), (c), (d), (e), (f), (g) and (h) the endurance characteristics of the HfAlAO thin films (2.17% Al and 4.2% La) under 85, 125, 150 and 175℃, respectively. (i) The effect of applied voltages and temperature on the device endurance. (j) The impact of temperature and cycles on the device polarization.

As shown in Fig. S14(a)-(h), the HfAlAO (2.17% Al and 4.2% La) shows similar trend with the HfLaO thin films with the increase of the applied voltage. However, the HfAlAO thin films show also excellent thermal endurance. When the test temperature is at 175 ℃，the endurance cycles can reach 10^6^ under applied voltages of 2.5 V. Fig. S14(i) shows the endurance cycles shows rapidly descend over applied voltages of ~3.7V. As shown in Fig. S14(j), the polarization of HfAlAO thin films is minimum of about 22 μC/cm^2^ at test temperature of about 175 ℃.

**S15 the thermal endurance of HfAlO thin films under 85, 125, 150 and 175 ℃, respectively.**


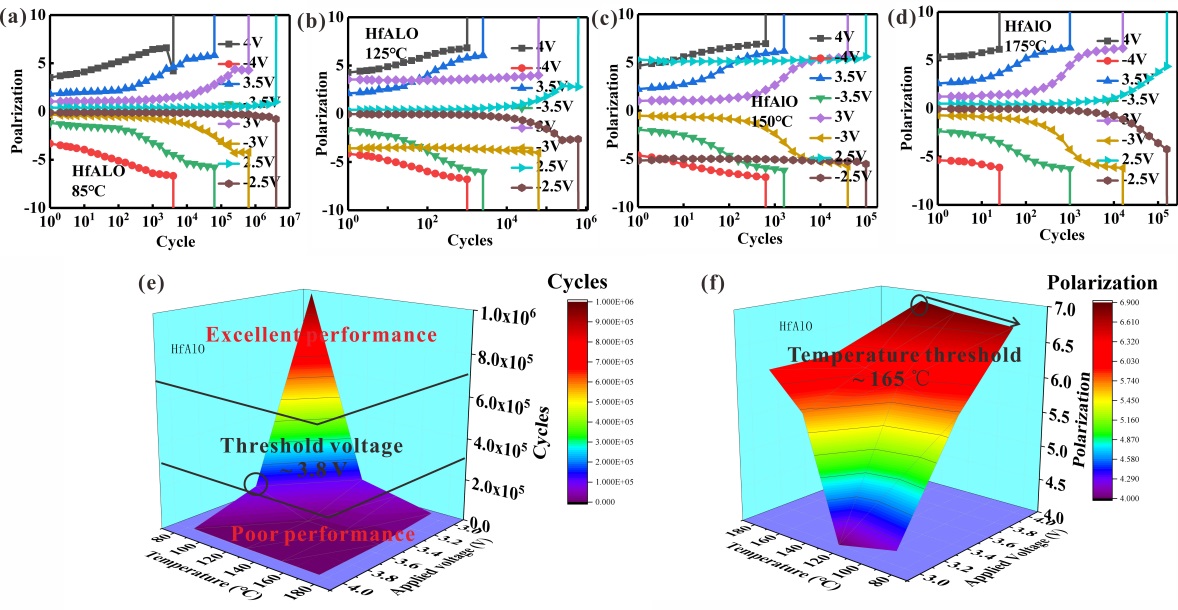


Fig. S15 (a), (b), (c) and (d) the thermal endurance characteristics of HfAlO thin film under 85, 125, 150 and 175 ℃. (e) The effect of the temperature and applied voltages on the endurance characteristics. (f) The impact of the temperature and applied voltages on the device polarization.

For the HfAlO thin films, Fig. S15(a)-(d) show that the endurance characteristics represent the obvious degradation with the increase of test temperature. When the test temperature reaches the 175 ℃，the endurance cycle is about 10^5^ under applied voltage of 2.5 V. As shown in Fig. S15 (e), the endurance cycles is dramatic decline over the applied voltage of about 3.8 V. Meanwhile, Fig. S15 (f) shows that the HfAlO devices display maximum polarization of about 7 μC/cm^2^ at ~165℃.

**S16 The failure mechanism of film damages under high temperature.**


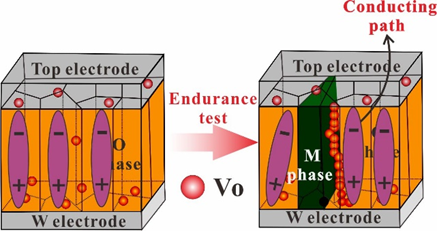


Fig S16 The failure mechanism of film damages under high temperature

As shown in Fig S16, the oxygen vacancy filament is easily formed under high temperature, which restricts the domain flipping. This may be the reason of the ferroelectric degradation.

**S17 Hafnium-based ferroelectricity devices for flexible application**


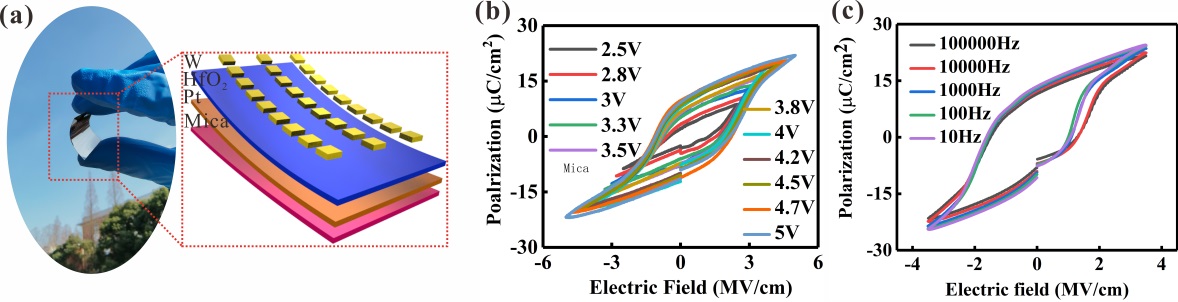


Fig.S17 (a) Optical image and structural schematic diagram of the flexible HfALAO ferroelectric devices. The P-E curves of the flexible devices (b) under different voltages and (c) different frequent.

Fig. S17 (a) shows the optical image and device structure diagram of the prepared devices, illustrating the flexibility and bendability of the prepared devices. The P-E hysteresis curves of the flexible HfALAO devices are shown in Fig. S17 (b). With the increase of sweeping voltage, the larger hysteresis is observed. Inversely, the remanent polarization displays degradation with increasing of the sweeping frequency, as shown in Fig. S17 (c). Those properties and trends are similar with the W/HfALAO/W/Si devices, which lay a good foundation for the flexible application of the Hf-based ferroelectric devices.

**S18 The structure of orthorhombic HfO_2_ without defect and with La-related and Al-related defect.**


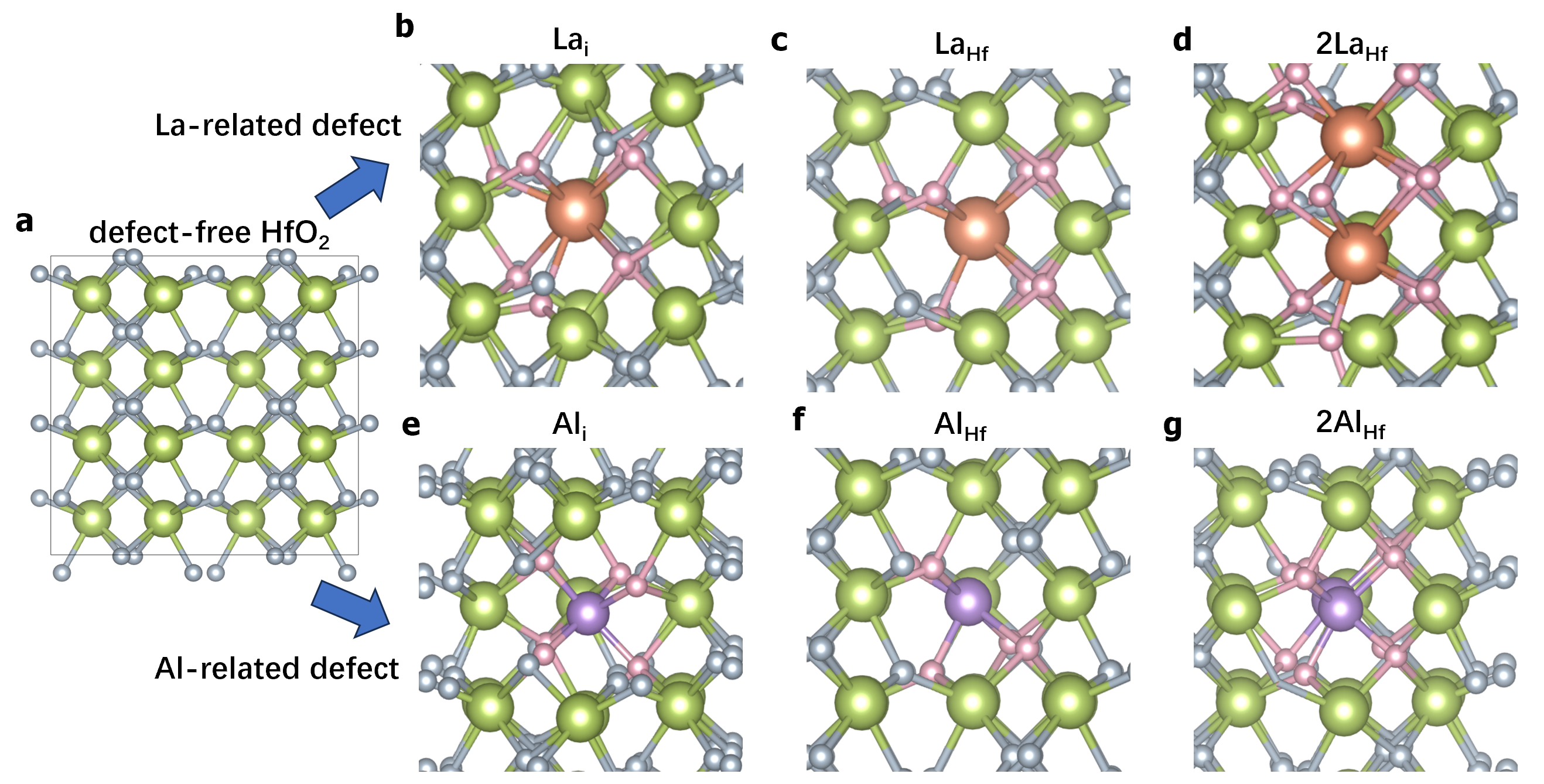


Figure S18: The structure of orthorhombic HfO_2_ (a)without defect and with (b)La_i_, (c)La_Hf_, (d)2La_Hf_, (e)Al_i_, (f)Al_Hf_ , (g)2Al_Hf_ defect. Green spheres represent Hf atoms, gray sphere represents O atoms, purple spheres represent Al atoms and orange sphere represents La atoms. In particular, the O atoms bonded to La and Al atoms are rendered pink.

The defect structure used during the calculation are shown in Fig.S18. In defect-free HfO­_2_, Hf atoms are ordered with a coordination number of 7. In defective structure with La_Hf_, the coordination number of La remains 7. The introduction of La_Hf_ defect won’t cause large lattice distortion, that’s why the formation energy of La_Hf_ defect is low. However, due to the large size of La atom, the introduction of La_i_ and 2La_Hf_ causes large distortion, so the formation of Lai and 2La_Hf_ defect is much higher than that of La_Hf_. Compared with La, Al atom is much smaller, so the Al_i_ and 2Al_Hf_ defect are easier to form than Lai and 2La_Hf_ defect.

**S19 Fermi level and formation energy of oxygen vacancy under different doping condition in La and Al co-doped system.**

Fig.S19: (a) The Fermi level and (b) the formation energy of V_O1_, (c) the formation energy of V_O2_ at different Al and La doping content. V_O1_ represents 3-coordination oxygen vacancy and V_O2_ represents 4-coordination oxygen vacancy.

Fig.S19 visually shows the variation of Fermi level and $V_{O}^{2+}$ defect formation energy with different doping content. As the La content increases form low La content to high La content, the Fermi level decreases. While with the Al content varies from the intermediate content to high content, the Fermi level raises. The trend of $V_{O}^{2+}$formation energy is consistent with that of Fermi level. With La content increasing, the $V_{O}^{2+}$formation energy drops constantly, but as the Al content increases from intermediate content to high content, the $V_{O}^{2+}$formation energy elevates.

**S20 Density of oxygen vacancy under different doping condition in La and Al co-doped system**

Fig.S20: (a) The density of $V_{O1}^{2+}$ and (b) the density of $V_{O2}^{2+}$ at different Al and La doping content. V_O1_ represents 3-coordination oxygen vacancy and V_O2_ represents 4-coordination oxygen vacancy.

Fig.S20 shows the density of $V_{O1}^{2+}$ and $V_{O2}^{2+}$ varied with different doping content. In La and Al co-doped system, the density of oxygen vacancy is higher at low or intermediate Al content and intermediate or high La content. Comparing the $V_{O}^{2+}$density at different Al content, it reaches the peak at intermediate Al content. And comparing the $V_{O}^{2+}$density at different La content, it increases constantly with the increase of La content.

**S21 EDS mapping of Hf, O and W**


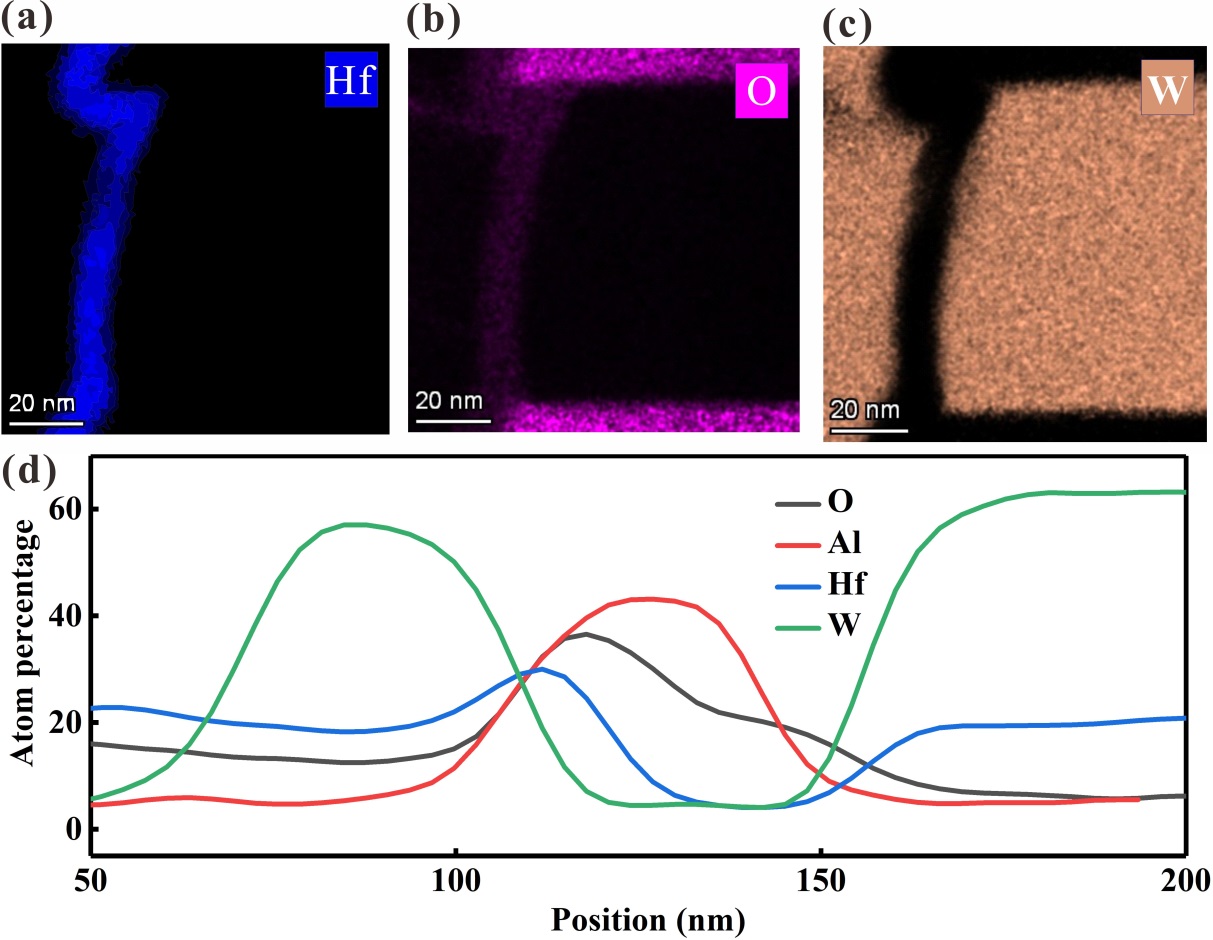


Fig. S21 EDS mapping image of the W/HfAlAO/W devices.

Fig . S21 shows that the energy dispersive X-ray spectroscopy (EDS) image of the ten layer vertical structure, confirming the uniform HfO_2_ thin films.

**S22 Synaptic properties of hafnium-based thin films**


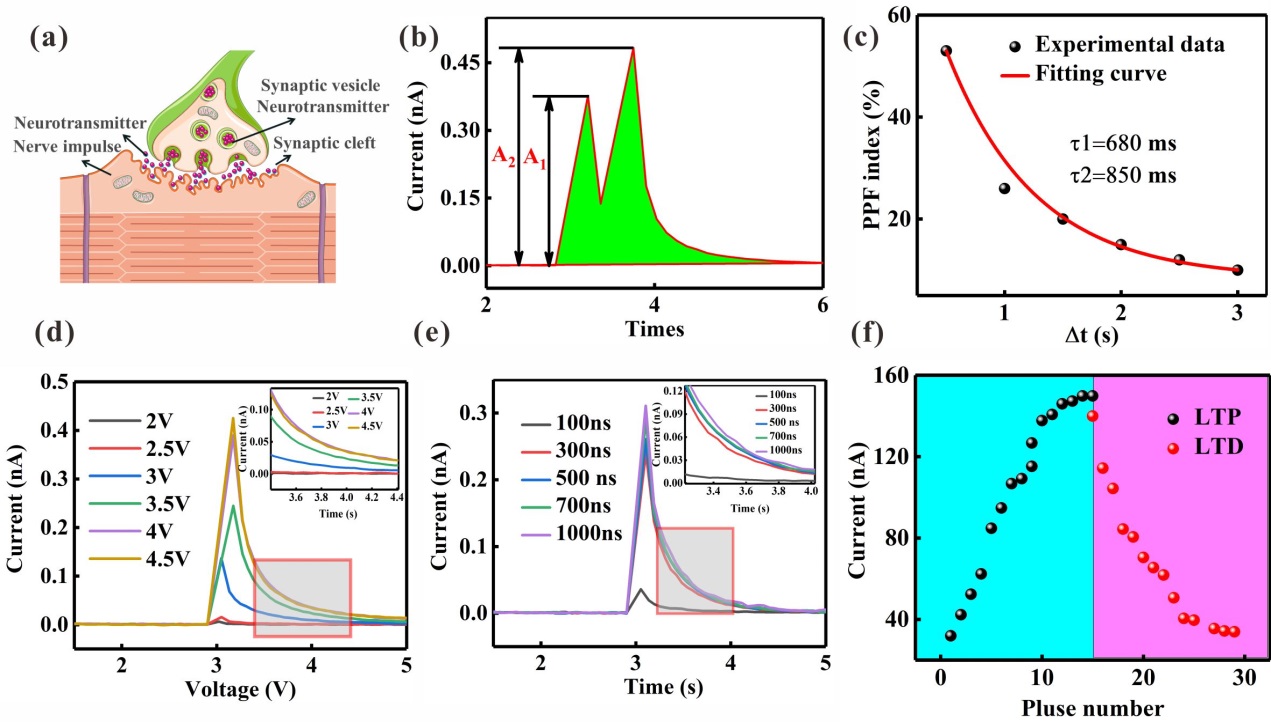


Fig. S22. (a) Schematic diagram of biological synaptic information transmission, (b) a pair of pulse is to simulate the PPF process. (c) The PPF index is a function of the time interval, synaptic properties of HfALAO devices. the relationship between current and (d) pulse amplitude (pulse width=100 ns) and (e) pulse width (pulse amplitude=3V), (f) LTP/LTD characteristics simulated by the applying continuous pulse.

The plasticity of bio-synapse could be emulated by applying pulses to pre-terminal of artificial synapses, as shown in Fig. S22(a). The short or long-term memory characteristics are defined by the maintained time of changed synaptic weights. The paired pulse facilitation (PPF) is an important manifestation of short-term plasticity. By applying two pulses at a time interval of 500ms, Fig. S22 (b) shows that the postsynaptic current response of subsequent pulses is obviously improved. The PPF index is used to characterize the relationship between the reinforcement effect of postsynaptic current pulse and the time interval. The PPF index can be fitted using a double exponential function.

$PPF=A+B_{1}exp(\frac{-t}{{}_{1}})$+$B_{2}exp(\frac{-t}{{}_{2}})$

Where t is the time interval, B1 and B2 are the origin facilitation magnitudes of the response current phases. The decay process of the PPF index is shown in Fig. S22(c). In addition to short-term plasticity, long-term plasticity is also important for the learning and memory, as shown in Fig.S22(d) and (e). With the pulse amplitude increasing from 2 to 4.5V, the excitatory Postsynaptic Current (EPSC) of HALAO thin films increase from 0.006 to 0.52 nA, as shown in Fig. S22(d). Similarly, Fig. S20(e) shows the larger response current is also obtained under the larger pulse width. Fig. S22 (f) shows the realization process of long-term plasticity (LTP) and long-term depression (LTD). By applying 15 continuous pulse of amplitude of -4V and pulse width of 100ns, the response current gradually increases. The LTD process is realized by applying pulse of the +4 V.

**S23 Image recognition of hafnium-based devices**


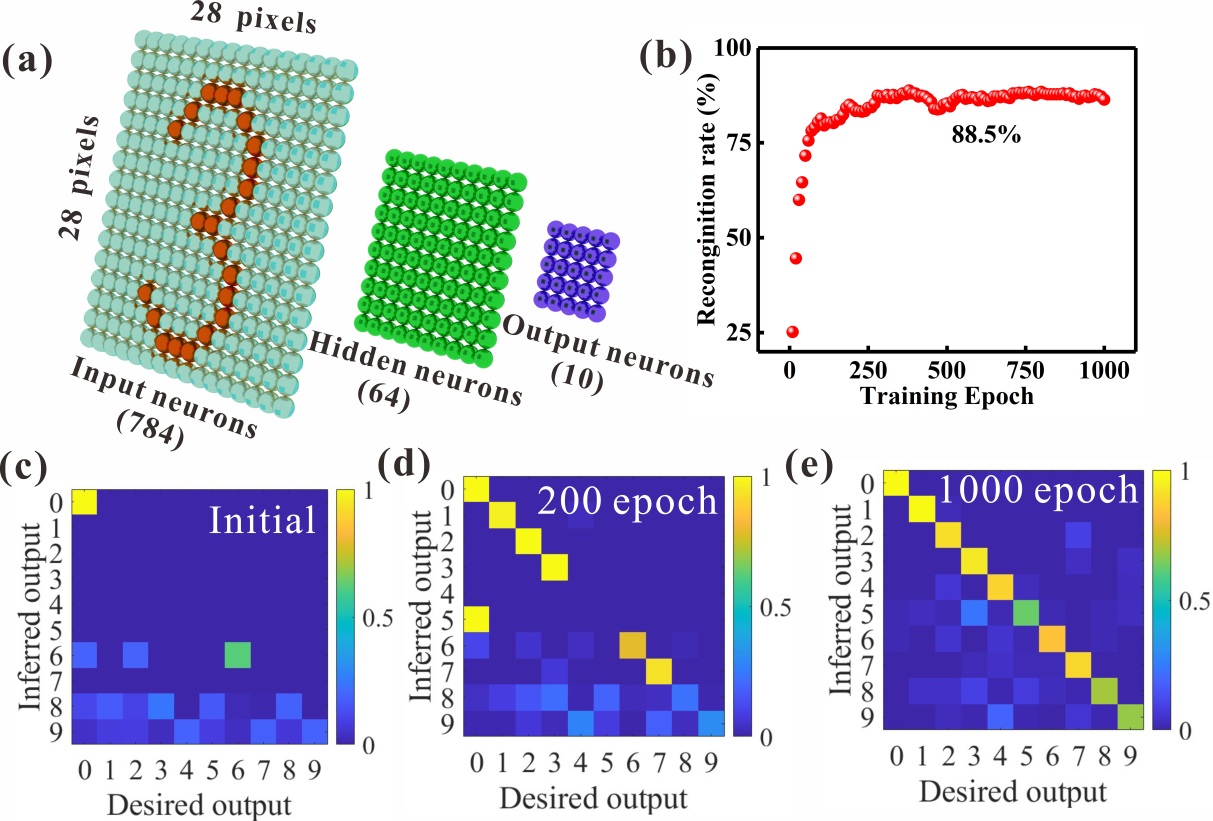


Figure. S23(a) the schematic diagram of three-layer artificial neural network for digital recognition (b) the recognition rate and (c)-(e) confusion matrix during 1000epochs learning training.

Base on the LTP/LTD behavior of HfALAO devices, the MNIST pattern recognition is simulated by three-layer arrays including input layer (784 neurons), hidden layer (64 neurons) and output layer (10 neurons), as shown in Fig. S23(a). The images of number “3” are used to simulate recognition of artificial neural network (ANN). Through the ANN training of 1000 epoch, the recognition rate is 88.5% (see the Fig. S23(b)). The training process of the recognition is a gradual learning process for the neural network, which reflects through the color of the diagonal line in the confusion matrix (see the Fig.S23 (c)). With increasing of the training times, the diagonal color appears the yellow in the output matrix, as shown in Fig. S23(d). The diagonal color is finally changed into the yellow in the output matrix, and Fig. S23(e) shows all patterns (“0-9”) are successfully identified, which demonstrates the great potential of the HfALO artificial synapses in information processing.
